# Secretome Analysis of Inductive Signals for BM-MSC Transdifferentiation into Salivary Gland Progenitors

**DOI:** 10.3390/ijms21239055

**Published:** 2020-11-28

**Authors:** Mahmoud Mona, Firas Kobeissy, Yun-Jong Park, Rehae Miller, Wafaa Saleh, Jin Koh, Mi-Jeong Yoo, Sixue Chen, Seunghee Cha

**Affiliations:** 1 Oral and Maxillofacial Diagnostic Sciences, University of Florida College of Dentistry, Gainesville, FL 32610, USA; mmona@dental.ufl.edu (M.M.); RMiller2@dental.ufl.edu (R.M.); 2 Oral Biology, University of Florida College of Dentistry, Gainesville, FL 32610, USA; 3 Department of Emergency Medicine, McKnight Brain Institute, University of Florida, Gainesville, FL 32610, USA; firasko@ufl.edu; 4 Division of Viral Products, Center for Biologics Evaluation and Research, Food and Drug Administration, Silver Spring, MD 20993, USA; Yun-Jong.Park@fda.hhs.gov; 5 Oral Medicine and Periodontology Department, Faculty of Dentistry, Mansoura University, Mansoura 35516, Egypt; wafaasaid@mans.edu.eg; 6 Interdisciplinary Center for Biotechnology Research, University of Florida, Gainesville, FL 32610, USA; jinkoh@ufl.edu (J.K.); schen@ufl.edu (S.C.); 7 Department of Biology, Clarkson University, Potsdam, NY 13699, USA; myoo@clarkson.edu; 8 Department of Biology, Genetics Institute, University of Florida, Gainesville, FL 32610, USA; 9 Center for Orphaned Autoimmune Disorders, University of Florida College of Dentistry, Gainesville, FL 32610, USA

**Keywords:** mouse bone marrow-derived stem cells (mMSC), co-culture, secretome, salivary glands, Sjögren’s syndrome, salivary progenitors, transdifferentiation

## Abstract

Severe dry mouth in patients with Sjögren’s Syndrome, or radiation therapy for patients with head and neck cancer, significantly compromises their oral health and quality of life. The current clinical management of xerostomia is limited to palliative care as there are no clinically-proven treatments available. Previously, our studies demonstrated that mouse bone marrow-derived mesenchymal stem cells (mMSCs) can differentiate into salivary progenitors when co-cultured with primary salivary epithelial cells. Transcription factors that were upregulated in co-cultured mMSCs were identified concomitantly with morphological changes and the expression of acinar cell markers, such as α-amylase (AMY1), muscarinic-type-3-receptor(M3R), aquaporin-5(AQP5), and a ductal cell marker known as cytokeratin 19(CK19). In the present study, we further explored inductive molecules in the conditioned media that led to mMSC reprogramming by high-throughput liquid chromatography with tandem mass spectrometry and systems biology. Our approach identified ten differentially expressed proteins based on their putative roles in salivary gland embryogenesis and development. Additionally, systems biology analysis revealed six candidate proteins, namely insulin-like growth factor binding protein-7 (IGFBP7), cysteine-rich, angiogenetic inducer, 61(CYR61), agrin(AGRN), laminin, beta 2 (LAMB2), follistatin-like 1(FSTL1), and fibronectin 1(FN1), for their potential contribution to mMSC transdifferentiation during co-culture. To our knowledge, our study is the first in the field to identify soluble inductive molecules that drive mMSC into salivary progenitors, which crosses lineage boundaries.

## 1. Introduction

Acinar and ductal cells in the salivary glands are fundamental units of saliva production and s secretion. Saliva is critical for food digestion, taste, and lubrication, as well as oral homeostasis and immunity [1]. Secretory dysfunction by radiotherapy, surgery, chemotherapy, or Sjögren’s syndrome results in life-disrupting pathological outcomes that involve swallowing difficulty, loss of taste, troubled speech, dental caries, and candida infection [2]. Dry mouth may also be caused by medications, diabetes, chronic kidney disease, psoriasis, and many other conditions [3,4,5,6].

Current treatment options are limited to palliative approaches, such as saliva substitutes and systemic secretagogues (pilocarpine or cevimeline) that stimulate saliva secretion from the residual acinar cells [7,8]. Therefore, a novel regenerative therapy using stem cells offers hope for a long-term cure in patients [9]. Salivary regenerative methods are focused on cell-based approaches, which necessitate the identification of potential progenitors or resident stem cells in order to substitute damaged ductal and acinar cells. Several studies have reported the presence of stem cell populations in mouse, rat, and human salivary glands. However, the scarcity of these cells has raised a challenge [10,11]. Thus, utilization of a more abundant source of stem cells, such as bone marrow- and adipose-derived stem cells, has been explored to overcome this challenge.

Mesenchymal stem cells (MSCs) are multipotent stem cells with the ability to differentiate into many cell types such as chondrocytes, adipocytes, osteoblasts, and salivary epithelial cells [12]. MSCs have been investigated for in vitro and in vivo experimental studies and clinical studies in various conditions due to their anti-inflammatory effects, low immunogenicity, and potential to repair damaged tissues [12,13,14]. They were also reported to have a vital role in regenerating human organs and tissues, such as bone marrow, brain, eye, intestine, lung, skin, adipose tissue, and dental pulp [15,16,17,18,19,20,21].

Interestingly, recent studies have shown that MSCs have therapeutic potential in the treatment of Sjögren’s syndrome. Intravenous injection of MSCs in mice with Sjögren’s syndrome-like disease resulted in an improvement of salivary flow rates as well as decreasing lymphocytic infiltrates and inflammatory cytokines in salivary gland cells [12,22]. Furthermore, it has been reported that injection of MSCs into the irradiated salivary glands of mice resulted in MSC transdifferentiation into acinar cells, increased saliva production, decreased apoptosis, and improved salivary gland weight [13,14,23]. Nevertheless, the process is hampered by the hostile microenvironment of the injured salivary glands [23,24]. The induction of MSCs at pre-transplantation might enhance their potential to differentiate, survive, and regenerate damaged salivary glands.

In order to identify endogenous MSC differentiation factors, we previously co-cultured mouse bone marrow-derived MSC (mMSC) with primary salivary gland cells (pSGC) in a transwell system over a period of 7 days. Starting at day 1 and throughout the course of co-culture, mMSCs adopted the round and cluster morphology of pSGCs and expressed salivary gland markers including α-amylase (AMY1), aquaporin-5 (AQP5), and muscarinic type 3 receptor (M3R), which was detected by isobaric tags for relative and absolute quantitation (iTRAQ) proteomics [25,26].

In this study, we applied a temporal high-throughput liquid chromatography with tandem mass spectrometry (LC-MS/MS) secretomics analysis and an advanced bioinformatics platform to the conditioned media collected from our co-culture system. The main purpose of our study was to identify soluble factors that served as exogenous inductive signals for mMSC transdifferentiation into salivary progenitors.

## 2. Results

### 2.1. Secretome Data Analysis and Gene Ontology (GO) Classification

**Figure 1:**
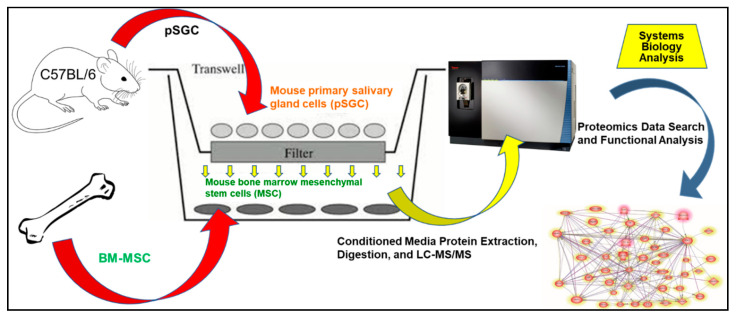
Experimental workflow. The workflow summarizes the various steps involved in our approach. (1) Primary salivary gland cell (pSGC) isolation from 4 week old male C57BL/6 mice; (2) co-culture of mouse bone marrow-derived mesenchymal stem cells (mMSC) and pSGC for 1, 3, 5, and 7 days; (3) conditioned media collection from each time point, LC-MS/MS processing of control samples (i.e., media alone, media from the mMSC culture, and media from the pSGC culture) and the experimental samples (conditioned media samples from co-culture of mMSC and pSGC); and (4) secretome data acquisition and systems biology analysis.

**Figure 2:**
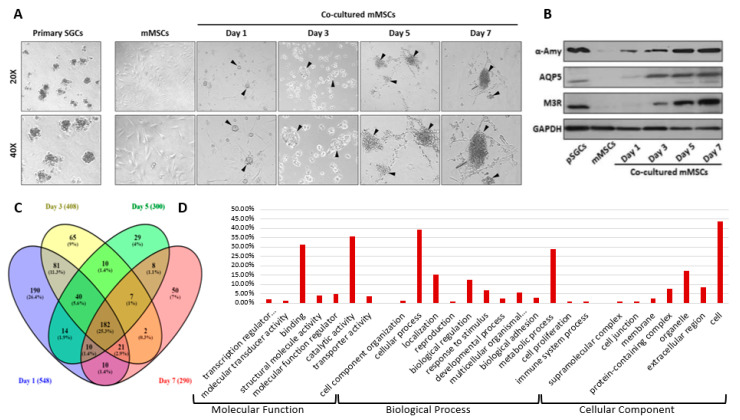
Morphological changes of mMSCs in the co-culture system and characterization of the secreted molecules detected in the conditioned media. (**A**) Microscopic images of pSGCs in the left panel and co-cultured mMSCSs in the right panels at days 1, 3, 5 and 7. All images are shown at a 20X (upper panel) and 40X (lower panel) magnification. Aggregated islets of mMSCs are present on day 1 of co-culture, resembling the islet-like appearance of pSGCs. (**B**) Salivary gland markers such as α-AMY, AQP5, and M3R were confirmed by western blotting. (**C**) A Venn diagram showing the number and percentage of secreted proteins detected at each collection time point. (**D**) A total of 548 secreted proteins on day 1 were assigned to 26 functional groups using Gene Ontology (GO). The three main categories consist of molecular function, biological process, and cellular component. The calculated percentages on the Y-axis were based on the proportion of the identified proteins in each gene set (GO: http://geneontology.org/docs/go-enrichment-analysis/). Figure 2A,B, reprinted from refs. [25,26], respectively.

In our previous studies, we found that mMSCs differentiated into salivary progenitor cells upon co-culturing mMSCs with pSGCs that had no cell-to-cell contact [25,26] (Figure 1). mMSC clusters resembling pSGCs while in co-culture were confirmed by light microscopy, as shown in Figure 2A. Furthermore, we observed that fibroblast-like mMSCs have altered their shape into round epithelial-like cells similar to pSGCs within 24 h of co-culture. These findings were confirmed by the expression of salivary gland markers in co-cultured MSCs, such as AMY1, AQP5, and M3R (Figure 2B). On the other hand, negative control (mMSC culture without pSGCs) did not undergo those changes and maintained their spindle shape-like appearance of mMSCs (Figure 2A).

We further performed LC-MS/MS to detect exclusively secreted proteins from the cells into the co-culture media, compared to the control media samples collected from mMSC culture, pSGC culture, or media alone. Our assay resulted in a total of 2798 proteins detected in all conditions. However, we found 201 proteins in the mMSC media and 130 proteins in the pSGC culture media. Therefore, we narrowed down the number of proteins to 548, 408, 300, and 290 proteins on days 1, 3, 5, and 7, respectively, after subtracting proteins detected in the controls (mMSC media, pSGC culture media, and media only) from all proteins detected in co-culture (Figure 2C). Since morphological and molecular changes in the transdifferentiated mMSCs were mainly observed within 24 h of co-culture [25,26], we primarily focused on our analysis for day 1. The current proteomics data have been deposited into the ProteomeXchange Consortium [27] via the MassIVE partner repository with the data set identifier PXD016181 and MSV000084544.

As shown in the Venn diagram (Figure 2C), 182 proteins were newly secreted throughout the 7-day co-culture. Interestingly, compared to all time points, day 1 showed the highest number of newly secreted proteins (190 proteins, 26.4%) (Figure 2C). To further categorize various regulatory factors on day 1, GO were assigned to 548 proteins based on their putative functions, which included molecular function, biological process, and cellular component (http://www.geneontology.org). As shown in Figure 2D, “binding” and “catalytic” activities were the most common types of molecular functions, and “cellular” and “metabolic” processes were the most activities present at day 1. Even though a significant percentage of the collected secretome was composed of extracellular and membranous proteins, intracellular proteins were also detected, presumably due to the presence of dead cells during the culture. We further analyzed our secretome detected at day 1 for their biological functions in the extracellular compartment.

### 2.2. Protein Clusters and Cellular Function Analysis of Newly Secreted Proteins in the Conditioned Media

Of the 548 proteins present at day 1 in the conditioned media, we confirmed 57 proteins to be associated with the extracellular compartment by searching the high-throughput global database with the Pathway Studio^®^ software. These 57 proteins in the media were listed with their UniProt numbers and gene names in Table 1. All resulted proteins were further investigated for their involvement in development-related cell processes, such as cell proliferation, cell differentiation, and cell colony formation. As a result, 21 proteins were found to be involved in cell differentiation or associated with cell colony formation (Table 2).

### 2.3. Pathway Enrichment of Newly Expressed Proteins in the Conditioned Media

**Figure 3:**
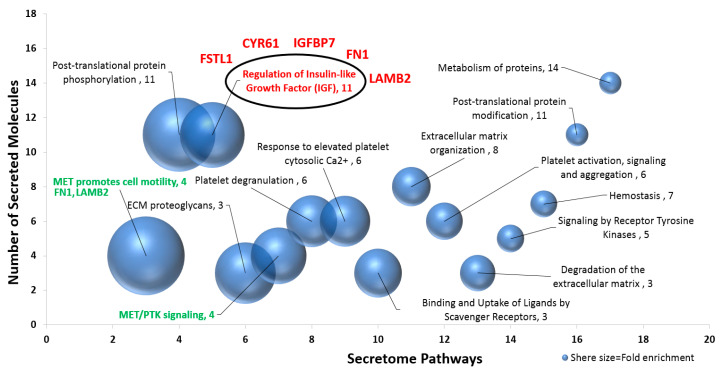
Pathway enrichment of newly secreted proteins detected in co-cultured MSCs at day 1. Fifty-seven newly detected secreted proteins in the conditioned media of differentiating MSCs were categorized by their predicted pathways (X-axis). In the bubble chart, the Y-axis represents the number of proteins involved in each pathway, and the size of the sphere represents the enrichment fold calculated by the Fisher’s exact test (*p* < 0.05). PANTHER software was utilized for this analysis (http://www.pantherdb.org/). Proteins in green are related to the mesenchymal epithelial transition pathway (MET), and proteins in red are members of the insulin-like growth factor (IGF) pathway. Fibronectin 1(FN1) and laminin, beta 2 (LAMB2) belong to both pathways.

**Figure 4:**
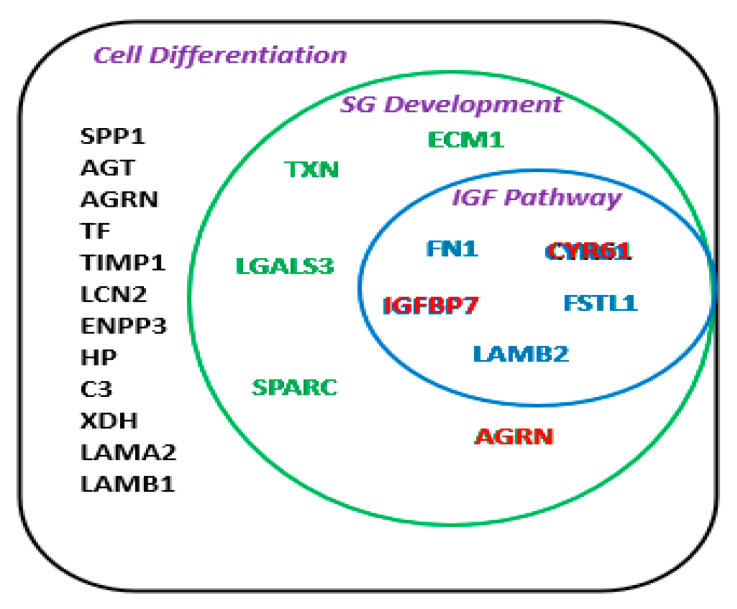
Secretory proteins involved in cell differentiation, which were identified in the conditioned media of mMSC-pSGC co-culture at day 1. The secretome contained 21 proteins that are known to play a role in cell differentiation. Of these 21 proteins, a group of ten secretory proteins was found to be expressed during mouse salivary gland (SG) development (green circle), five proteins belonged to the IGF pathway (blue circle), and three proteins contained a growth factor domain (red).

We categorized the identified proteins by their possible pathways, involved molecular functions, and GO enrichment (Figure 3). The sphere plot categorizes these molecules by each pathway and scores them by the number of molecules involved in a pathway in addition to their level of enrichment. Sphere size represents the fold of enrichment of these pathways compared to the global gene database. Enrichment values in the GO mouse library were calculated by the Fisher’s exact test (*p* < 0.05). Evidently, pathways associated with growth factors such as insulin-like growth factor (IGF) and mesenchymal-epithelial transition (MET) were of the most enriched in our dataset (Figure 3). The IGF-related pathway accounts for five of ten proteins expressed during salivary gland development (Figure 4).

The level of evidence of protein involvement in cell differentiation was presented as the number of times it was reported in the literature. For example, Agrin (AGRN) was reported 90 times for its role in multiple cell-type differentiation and tissue development (Table 2). Of interest, we found that 10 out of 21 (47.6%) secreted proteins with a putative role in differentiation were also expressed in the mouse submandibular and parotid glands during their embryonic development. They are known to be expressed in the developing gland from E11 until adulthood, according to the National Institute of Dental and Craniofacial Research (NIDCR) Salivary Gland Map database (http://sgmap.nidcr.nih.gov/sgmap/sgexp.html). Table 3 further describes the expression pattern of those ten proteins in the salivary glands and cells. The proteins include fibronectin 1 (FN1), AGRN, Osteonectin (SPARC), lectin, galactoside-binding, soluble, 3 (LGALS3), cysteine-rich, angiogenic inducer, 61 (CYR61), insulin-like growth factor binding protein 7 (IGFBP7), thioredoxin (TXN), follistatin-like 1 (FSTL1), and extracellular matrix protein 1 (ECM1). Their expression level also varied by the location in the glands (Table 3).

### 2.4. Intracellular and Extracellular Interactome

In a previous study, we identified a list of 28 intracellular proteins putatively involved in mMSC transdifferentiation by iTRAQ [25]. Putative pathways of interest associated with our newly discovered secreted molecules are summarized in Table S1. Using the Pathway Studio^®^ for proteomics analysis, we identified all the potential interactions between our groups of secreted proteins and differentially expressed intracellular proteins in co-cultured mMSCs that were previously identified by iTRAQ, as shown in Supplementary Materials Figure S1 and Table S2. Figure S1 depicts the network of predicted association between the secreted molecules (yellow halos) and the intracellular molecules (green halos). Among the secreted molecules, ten proteins with red halos represent the extracellular molecules that are known to be associated with salivary gland development in mice according to the NIDCR Salivary Gland Map database. The thicker the interconnecting line, the stronger the association (Figure S1).

## 3. Discussion

Numerous approaches have been proposed to restore impaired salivary gland function, which is primarily caused by radiation therapy or autoreactive immune cells in Sjögren’s syndrome. Current methods are limited to preventative and palliative measures, such as minimizing radiation dosage to surrounding tissues by intensity-modulated radiation therapy [28], reducing radiation damage by scavenging radicals, and using immunosuppressants, or sialagogues in case of the autoimmune condition [29]. In recent years, novel approaches such as gene transfer [30] or stem cell regeneration [31,32] have received great attention. However, the hostile and altered microenvironment due to cellular damages may hinder proper differentiation of transplanted stem cells or resident stem cells in the glands [33,34,35,36]. Therefore, understanding the underlying molecular mechanisms of stem cell differentiation to a desired cell type would enhance the success of reprogramming stem cells ex vivo followed by the transplantation for terminal differentiation in situ.

MSCs can differentiate into a variety of cell types including osteoblasts, chondrocytes, myocytes, and adipocytes [37]. They are responsible for a lifetime of tissue remodeling and repair [38]. As stem cells usually require signals from their microenvironment for terminal differentiation, identifying intrinsic and extrinsic signals for stem cell differentiation is critical to facilitate the efficacy of cell-based therapies. Yet, the molecular inductive signals for MSC transdifferentiation into salivary progenitors have not yet been discovered. We previously confirmed the expression of salivary gland marker proteins, such as AMY, M3R, AQP5, and CK19, in co-cultured mMSCs and identified intrinsic transcription factors that were upregulated in the differentiating MSCs [25,26].

Shotgun proteomics, also known as the bottom-up proteomics technique, has been widely used for identifying proteins in stem cell studies using a combination of HPLC with mass spectrometry [39,40]. By employing this technique for the study, we identified novel proteins in the conditioned media when pSCGs and mMSCs were co-cultured in a transwell system without any cell-to-cell contact. We hypothesized that inductive signals in the media mainly originated from pSGCs, resulting in the lineage-specific transdifferentiation of mMSCs. These signals (i.e., proteins) were shown to be related to cellular functions, such as binding, catalytic activities, and biological process regulation. Further analysis has shown that 21 of 57 proteins (36%) are involved in developmental processes, such as differentiation. Of those 21 proteins, ten were found to be expressed at various times during mouse salivary gland development (i.e., FN1, CYR61, AGRN, SPARC, LGALS3, CYR61, IGFBP7, TXN, FSTL1, and ECM1), according to the NIH/NIDCR Salivary Gland Map database. Notably, the amino acid sequences of three proteins, CYR61, IGFBP7, and AGRN, contain a growth factor domain, emphasizing their possible role in cell differentiation and growth.

FN1 is a soluble glycoprotein expressed in the developing salivary gland mesenchyme. It is involved in cell adhesion and migration processes including embryogenesis and wound healing [32,33,34,35,36]. It has been also reported for its role in dental pulp differentiation [41]. FN1, in addition to collagen I and II, upregulated in pancreatic exocrine acinar cells corresponding to the overexpression of a protein named muscle, intestine and stomach expression 1 (MIST-1) [42]. It is important to note that our previous study revealed that MIST-1, a basic helix-loop-helix transcription factor for AMY1 expression, was upregulated in co-cultured MSCs [25,26,43]. Additionally, MIST1 was found to be an upstream key regulator of FN1 [44] and other transcription factors like TCF3 [45] and PTF1A [46]. Similarly, FN1 was shown to be a downstream target for several intracellular proteins, such as SOD2 [47] and cofilin 1(CFL1) [48]. In addition, FN1 and CYR61 regulate each other, which is suggestive of a closed loop association [49,50]. It is also reported that IGFBP7 positively regulates FN1 expression in fibroblasts [51,52]. Therefore, the functional roles of MIST1 in the regulation of FN1 and other identified factors in our current study will be further investigated and confirmed in our next study. The detailed list of putative pathways involving extracellular proteins is presented in Table S1.

On the other hand, AGRN is not associated with a specific cell type, but expressed in all cell types during salivary gland embryogenesis. It contains an epidermal growth factor domain and is found to be required for post- and pre-synaptic differentiation in the neuromuscular junction [53,54,55,56,57,58,59,60]. In addition, AGRN has also shown to enhance cartilage differentiation by upregulating SRY-Box 9 (Sox9) transcription factor [61]. Interestingly, our detected extracellular proteins include another subtype of SOX receptor, QSOX1. QSOX1 gene expression plays an important role in growth regulation of human lung fibroblast [62], but its role in mMSC transdifferentiation is currently unknown.

FSTL1 is also known for its role in cell differentiation in lung epithelium [63,64] and heart mesenchyme [33]. In a recent study, the sonic hedgehog pathway, a signaling pathway required for proper cell differentiation, was impaired as a result of FSTL1 deletion [63]. Another identified molecule with a role in cell differentiation was CYR61. It is known to interact with integrin and heparin sulfate proteoglycan [65] to promote cell proliferation, adhesion, and differentiation [66,67,68,69]. It induces differentiation in multiple cell types and stimulates chondrogenesis [70,71,72], ontogenesis [68,73], and angiogenesis [74]. Interestingly, it contains a growth factor domain [75]. This could imply that CYR61 may play an unrecognized role in salivary gland stem cell differentiation and/or glandular development similar to other identified proteins in our current study.

Another growth factor-like molecule secreted by the pSGCs into the co-culture media is IGFBP7. It has a reported role in keratinocyte differentiation and regeneration of multiple tissues with a therapeutic effect in psoriasis [76,77]. It is also known to regulate hematopoietic stem cell differentiation [78]. Since it was secreted during co-culture, we hypothesize that IGFBP7 plays a role as inducer or co-inducer of the observed mMSC transdifferentiation. Another putative molecule detected is LGALS3, which has shown to be involved in angiogenesis of endothelial cell differentiation [79,80] and embryonic development [81]. Interestingly, LGALS3 is also known for its affinity for laminin. They together have shown to induce capillary formation in Matrigel^®^ [82]. Laminin protein subtypes were also detected in our co-culture media including alpha 2, beta 1 and beta 2 (LAMB2). They are also known to contribute to cell differentiation, muscle tissue development [83], and dentin formation in the case of LAMB2 [84]. Of the three subtypes detected, LAMB2 gene expression was also found in mouse submandibular gland tissue, according to the NIDCR database. Therefore, it is conceivable that LGALS3 and LAMB2 together may play a role in driving mMSC transdifferentiation into salivary progenitors in our co-culture system.

Furthermore, the pathway analysis of 21 secreted proteins involved in cell differentiation and salivary gland development showed an importance of the IGF pathway as it was highly enriched in our dataset. The pathway encompasses five of our ten putative proteins, CYR61, IGFBP7, FN1, FSTL1, and LAMB2. The IGF pathway plays a critical role in cellular growth and development [85]. Its activation has shown to induce the process of salivary cell differentiation and branching morphogenesis [86]. Interestingly, the IGF pathway activation regenerated ligated salivary glands and induced AMY1 secretion in mice [87,88]. Furthermore, targeted activation of the pathway has shown to promote glandular recovery after radiation therapy in patients [87], suggesting that activation of the IGF pathway may promote healing of damaged salivary glands.

Multiple pathways were associated with the newly discovered proteins in our secretome analysis. The most enriched pathways included PTK2 signaling, cell motility, and laminin interactions (Table S1). Several pathways pertained to molecular interaction, signaling, and IGF regulation. Furthermore, when comparing the association between the newly discovered extracellular molecules with the previously reported differentially expressed intracellular proteins, we found through Pathway Studios^®^ that many reported functional interactions between these groups. Of interest, transcription factors MIST1 (BHLHA15) and TCF3 activate multiple extracellular components identified in our secretome (Figure S1, Table S2). As mentioned earlier, future experimental confirmations of these interactions are critical for the identification of key regulators that are responsible for cellular differentiation of mMSCs observed in the co-culture system. In summary, we narrowed down our candidate molecules to six putative proteins of interest (FN1, CYR61, FSTL1, AGRN, IGFBP7, and LAMB2), which is summarized in Figure 4. These molecules are believed to promote communication between mMSCs and pSGCs. Interestingly, all but AGRN were known to be associated with the IGF pathway, implying that the transdifferentiation of mMSCs in co-culture may occur via the activation of the IGF pathway. Although our combinatorial proteomics approach, which discovered intrinsic transcription factors in our previous study and secreted, exogenous factors in our current study, has generated invaluable and extensive information on the molecular network governing mMSC differentiation into salivary progenitors for the first time, the identification of key master regulators for this process and downstream signaling pathways requires further investigation. Our effort to link those two sets of data was limited by the lack of biological and functional data that can confirm our current bioinformatics data. It is of our utmost priority to investigate how these identified molecules influence each other to drive mMSC transdifferentiation into salivary progenitors and whether these progenitors can exert improvement of secretion once they are transplanted in vivo. Our next targeted approaches will be critical to confirm the essential roles of our identified proteins in promoting the efficacy of MSC-based therapeutics for patients with damaged salivary glands.

## 4. Materials and Methods

### 4.1. Animals

As described in our previous article [25], we used 4–6 week old C57BL/6J male mice. They were maintained in a pathogen-free condition within the University of Florida Animal Care Facility. A total of 15 mice were utilized for co-culture, and the conditioned media samples were collected from co-culture for each time point. The University of Florida Institutional Animal Care and Use Committee (IACUC) has approved breeding and animal use (protocol #201807411 approved on 29 April 2018) The American Veterinary Medical Association guidelines were followed in euthanizing the mice by deep isoflurane anesthesia followed by the recommend cervical dislocation.

### 4.2. Mouse Bone Marrow-Derived Mesenchymal Stem Cell Culture

mMSCs were harvested from 8-week of C57BL/6 mice bone marrow. Animals were purchased from Life Technologies, Inc. The manufacturer assured a purity of >95% of a positive expression of a stem cell marker, such as CD29+, CD44+, CD34+, Sca1+. Moreover, the manufacturer confirmed the cell’s ability to differentiate in vitro into multiple cell types, such as osteocytes, adipocytes, and chondrocytes. We also confirmed stemness of the cells by western blotting with antibodies for Sca1, Thy1, and CK45 (data not shown). mMSCs were cultured in DMEM/F12 with 10% stem-qualified fetal bovine serum (FBS) and 1μg/mL of penicillin/streptomycin antibiotics, following the manufacturer’s recommendations. Cells were incubated in 5% CO2 at 37°C, and they were maintained under 80–90% confluence. In all our experiments, we used mMSCs with passages between 3 and 6 after thawing. pSGC isolation, purification, and culture were carefully performed to avoid contamination following a published protocol [89]. In brief, submandibular gland tissues excised from 4–6 week old male C57BL/6J mice were finely sliced. Hanks’ balanced salt solution (HBSS) containing 1% (*w*/*v*) bovine serum albumin (BSA) and collagenase II (0.25 mg/mL) (Life Technologies, Inc.) and CaCl_2_ (6.25 mM) at 37 °C for 40 min in a water-bath to further digest the glandular tissues. Later, cells were filtered through a 100 μm steel mesh and transferred to a 60 mm petri dish at about 1.3 × 10^6^ cells per plate. Epithelial cells, verified by AMY1 expression later on (data not shown), were concentrated in the center of the petri dish by manual rotation. We collected pSGCs and cultured them for 12 h in the serum-free Hepato-STIM media(BD BioCoat™) with 500 U/mL penicillin/streptomycin prior to co-culturing with mMSCs. Eventually, 3.0 × 10^6^ to 3.5 × 10^6^ cells yielded from a single submandibular gland.

### 4.3. Co-Culture of mMSC and pSGC

Our co-culture experiments used 6- or 24-well plates containing a 0.4 μm pore size polycarbonate membrane-based transwell insert (Millipore Millicell^®^ cell culture inserts, EMD Millipore, Billerica, MA, USA). mMSCs were seeded onto the collagen-coated lower chamber at a density of 1.0 × 10^4^ cells/cm^2^. Cells were incubated in Hepato-STIM media without serum for 12 h prior to experiments. After mMSCs attached to the bottom of the plate, pSGCs (6 × 10^4^ cells/cm^2^) were seeded onto the membrane of the upper transwell insert. Cells in the co-culture system were maintained at 37 °C and 5% CO_2_ for 7 days without replacing the media. The culture media samples from the mMSC and pSGC co-culture, mMSC culture, and pSGC culture were collected. These samples were spun down at 3000 rpm for 10 min at 4 °C in a table-top centrifuge to precipitate cell debris or any intact cells, and the supernatant was carefully collected into a 1.5 mL tube for storage at −70 °C until total of four biological replicates were prepared from 1, 3, 5, or 7 days of co-culture.

### 4.4. Protein Extraction, Digestion, and LC-MS/MS

The proteins were concentrated with Amicon Ultra 3kDA cutoff centrifugal filters (EMD Millipore Inc., Billerica, MA, USA). Protein digestion and liquid chromatography tandem mass spectrometry (LC-MS/MS) were conducted as previously described [90], but with minor changes. Each sample (5 μg protein digest) with 50 fmol of peptide retention time calculation mixture (PRTC; Pierce, Thermo Fisher Scientific, Grand Island, NY, USA) was loaded onto the LC-MS/MS system. The flow rate was 250 nl/min, and the gradient was equilibration with solvent A (0.1% formic acid), followed by a linear increase from 0% to 25% solvent B (0.1% formic acid, 99.9% acetonitrile) in 110 min, then ramping up to 98% B and stayed for 10 min, and final equilibration with solvent A for 30 min. The mass spectrometer scan range was 350 to 2000 m/z. Each survey scan was followed by up to 40 MS/MS scans of the most intense precursor ions in the linear ion trap. Preview mode was enabled, and dynamic exclusion was set for 15 s.

### 4.5. Proteomics Data Search and Analysis

The MS/MS spectra were analyzed by a thorough database search using Mascot (version 2.4), with considerations of biological modification and amino acid substitution against a UniProt mouse database (84,937 entries download on 15 May 2017) with decoy option. The search parameters were peptide tolerance at 10 ppm, MS/MS ion tolerance at 1 Da, peptide charge from 2+ to 6+, trypsin as the enzyme, carbamidomethyl (C) as fixed modifications, and oxidation (M) and phosphorylation (S, T, Y) as variable modifications. The false discovery rates of peptides and proteins were controlled under 1% and 5%, respectively. Scaffold (version Scaffold_4.2.1, Proteome Software Inc., Portland, OR) was used to validate MS/MS-based identifications. Peptide identifications were accepted if they passed > 80.0% probability by the Peptide Prophet algorithm [90] with Scaffold delta-mass correction. Protein identifications were accepted if they established > 95.0% probability, assigned by the Protein Prophet algorithm [91]. Proteins that contained similar peptides and could not be differentiated based on the MS/MS analysis alone were grouped to satisfy the principles of parsimony. The spectral count for each protein was calculated by an assigned peptide from that protein with high confidence. To determine differentially expressed proteins, normalized spectral abundance factor (NSAF) was used, and data distribution was confirmed by the distribution of PRTCs. Analysis of variance (ANOVA) was performed using JMP 13.2.0 (SAS Institute, Cary, NC, USA.)

### 4.6. Functional and Statistical Analysis

The Pathway Studio Software (version 11.0; Ariadne Genomics/Elsevier Inc., Rockville, MD, USA) was used for protein function analysis as previously described [91,92]. The significance of differential proteins was assessed by t-test of *p*-value ≤ 0.05 with fold change >1.5 or <0.5. “Subnetwork Enrichment Analysis” (SNEA) algorithm was used to obtain statistically relevant biological and functional pathways. Fisher’s statistical test is used by SNEA to ascertain if there are significant associations between two variables formed by a specific relationship. The algorithm uses one-sided Mann–Whitney U-Test to compare sub-network distribution to the background distribution and calculates a *p*-value for the statistical significance of the difference between the two distributions.

For GO analysis, the PANTHER software (Protein Analysis Through Evolutionary Relationships; http://www.pantherdb.org/genes/batchIdSearch.jsp) Version 14.1 and GO [93] were used for molecular functions and biological process categorization. GO level 3 filtering was used to identify unique protein changes during comparison analysis. To determine potential involvement of the differentially expressed proteins detected by LC-MS/MS in the developing salivary glands, mRNAs known to be expressed in the glands were examined by accessing the NIDCR Salivary Gland Map website (http://sgmap.nidcr.nih.gov/sgmap/sgexp.html).

## Figures and Tables

**Table 1:** Extracellular proteins present in the conditioned media at day 1, identified by the Pathway Studio.

No.	Protein Name	Gene Name	UniProt
1	Ceruloplasmin (ferroxidase)	CP	G3X8Q5_MOUSE
2	Transferrin	TF	Q542D9_MOUSE
3	Glucose-6-phosphate isomerase	GPI	G6PI_MOUSE
4	Aldo-keto reductase family 1, member B10 (aldose reductase)	AKR1B10	Q5U415_MOUSE
5	Ectonucleotide pyrophosphatase/phosphodiesterase 3	ENPP3	ENPP3_MOUSE
6	Galactosamine (N-acetyl)-6-sulfate sulfatase	GALNS	GALNS_MOUSE
7	Xanthine dehydrogenase	XDH	B2RUJ7_MOUSE
8	Superoxide dismutase 3, extracellular	SOD3	Q64466_MOUSE
9	Thioredoxin	TXN	THIO_MOUSE
10	Quiescin Q6 sulfhydryl oxidase 1	QSOX1	QSOX1_MOUSE
11	Lectin, galactoside-binding, soluble, 3	LGALS3	LEG3_MOUSE
12	Lectin, galactoside-binding, soluble, 3 binding protein	LGALS3BP	Q07797_MOUSE
13	Fibronectin 1	FN1	Q9Z1Z8_MOUSE
14	Basal cell adhesion molecule (Lutheran blood group)	BCAM	Q99K86_MOUSE
15	Gelsolin	GSN	Q3TGJ9_MOUSE
16	Agrin	AGRN	AGRIN_MOUSE
17	Elastin microfibril interfacer 1	EMILIN1	Q3U254_MOUSE
18	Secreted protein, acidic, cysteine-rich (osteonectin)	SPARC	Q5NCU4_MOUSE
19	Collagen, type I, alpha 2	COL1A2	Q3TP88_MOUSE
20	Collagen, type VI, alpha 3	COL6A3	O88493_MOUSE
21	Thrombospondin 4	THBS4	B2RTL6_MOUSE
22	Hemicentin 1	HMCN1	D3YXG0_MOUSE
23	Angiopoietin 2	ANGPT2	ANGP2_MOUSE
24	Granulin	GRN	H3BJ90_MOUSE
25	Aminoacyl tRNA synthetase complex-interacting multifunctional protein 1	AIMP1	Q3UZG4_MOUSE
26	Cysteine-rich, angiogenic inducer, 61	CYR61	CYR61_MOUSE
27	Secreted phosphoprotein 1	SPP1	Q3UZY3_MOUSE
28	Angiotensinogen	AGT	Q8VCN0_MOUSE
29	Insulin-like growth factor binding protein 7	IGFBP7	Q3UFA6_MOUSE
30	Matrix metallopeptidase 2	MMP2	Q3UG07_MOUSE
31	Cathepsin B	CTSB	CATB_MOUSE
32	Lipocalin 2	LCN2	NGAL_MOUSE
33	Peroxiredoxin 4	PRDX4	PRDX4_MOUSE
34	Prosaposin	PSAP	Q3UE29_MOUSE
35	TIMP metallopeptidase inhibitor 1	TIMP1	TIMP1_MOUSE
36	Haptoglobin	HP	HPT_MOUSE
37	Laminin, beta 1	LAMB1	LAMB1_MOUSE
38	Chitinase, acidic	CHIA	CHIA_MOUSE
39	Complement component 3	C3	CO3_MOUSE
40	ISG15 ubiquitin-like modifier	ISG15	ISG15_MOUSE
41	Peroxidasin homolog (Drosophila)	PXDN	PXDN_MOUSE
42	Extracellular matrix protein 1	ECM1	Q9Z2R8_MOUSE
43	Sphingomyelin phosphodiesterase, acid-like 3B	SMPDL3B	ASM3B_MOUSE
44	ADP-dependent glucokinase	ADPGK	Q3UDS7_MOUSE
45	Insulin-degrading enzyme	IDE	F6RPJ9_MOUSE
46	Serpin peptidase inhibitor, clade C (antithrombin)	SERPINC1	ANT3_MOUSE
47	Protease, serine, 1 (trypsin 1)	PRSS1	E9QPR6_MOUSE
48	Transcobalamin II	TCN2	TCO2_MOUSE
49	Laminin, alpha 2	LAMA2	LAMA2_MOUSE
50	Laminin, beta 2	LAMB2	LAMB2_MOUSE
51	Follistatin-like 1	FSTL1	FSTL1_MOUSE
52	Family with sequence similarity 3, member D	FAM3D	FAM3D_MOUSE
53	Inter-alpha-trypsin inhibitor heavy chain family, member 4	ITIH4	ITIH4_MOUSE
54	Protease, serine, 22	PRSS22	Q7TML0_MOUSE
55	NHL repeat containing 3	NHLRC3	NHLRC3_MOUSE
56	Submandibular gland protein C	CP	B9EHK5_MOUSE
57	Submaxillary gland androgen regulated protein 3A	TF	TRFE_MOUSE

**Table 2:** List of 21 secreted proteins involved in cell differentiation as reported in the literature.

No.	Protein Name	Gene Name	UniProt	Level of Evidence *
13	Fibronectin 1	FN1	Q9Z1Z8_MOUSE	<100
27	Secreted phosphoprotein 1	SPP1	Q3UZY3_MOUSE	<100
28	Angiotensinogen	AGT	Q8VCN0_MOUSE	<100
16	Agrin	AGRN	AGRIN_MOUSE	90
57	Submaxillary gland androgen regulated protein 3A	TF	TRFE_MOUSE	76
18	Secreted protein, acidic, cysteine-rich (osteonectin)	SPARC	Q5NCU4_MOUSE	65
11	Lectin, galactoside-binding, soluble, 3	LGALS3	LEG3_MOUSE	57
35	TIMP metallopeptidase inhibitor 1	TIMP1	TIMP1_MOUSE	52
26	Cysteine-rich, angiogenic inducer, 61	CYR61	CYR61_MOUSE	46
29	Insulin-like growth factor binding protein 7	IGFBP7	Q3UFA6_MOUSE	23
32	Lipocalin 2	LCN2	NGAL_MOUSE	23
9	Thioredoxin	TXN	THIO_MOUSE	19
5	Ectonucleotide pyrophosphatase/phosphodiesterase 3	ENPP3	ENPP3_MOUSE	12
36	Haptoglobin	HP	HPT_MOUSE	10
39	Complement component 3	C3	CO3_MOUSE	10
51	Follistatin-like 1	FSTL1	FSTL1_MOUSE	10
42	Extracellular matrix protein 1	ECM1	Q9Z2R8_MOUSE	7
7	Xanthine dehydrogenase	XDH	B2RUJ7_MOUSE	6
49	Laminin, alpha 2	LAMA2	LAMA2_MOUSE	5
50	Laminin, beta 2	LAMB2	LAMB2_MOUSE	5
37	Laminin, beta 1	LAMB1	TRFE_MOUSE	4

* Level of evidence indicates the number of times reported in the literature.

**Table 3:** List of ten secreted proteins in the MSC co-culture media at day 1, which are known to be involved in cell differentiation and mouse salivary gland development.

No.	Protein Name	Gene Name	UniProt	Cell Type *		Epithelium *		Notes *
				Epithelium	Mesenchyme	End Bud	Duct	
13	Fibronectin 1	FN1	Q9Z1Z8_MOUSE		Y			Highly expressed early in development
16	Agrin	AGRN	AGRIN_MOUSE	Y	Y	Y	Y	Higher expression in epithelium
18	Osteonectin	SPARC	Q5NCU4_MOUSE	Y	Y	Y	Y	
11	Lectin, galactoside-binding, soluble, 3	LGALS3	LEG3 _MOUSE		Y			
26	Cysteine-rich, angiogenic inducer, 61	CYR61	CYR61_MOUSE	Y	Y		Y	2x in mesenchyme
29	Insulin-like growth factor binding protein 7	IGFBP7	Q3UFA6_MOUSE		Y			Expressed late in development
9	Thioredoxin	TXN	THIO_MOUSE	Y	Y	Y	Y	
51	Follistatin-like 1	FSTL1	FSTL1_MOUSE	Y	Y	Y	Y	3X in mesenchyme and 2X more in duct
42	Extracellular matrix protein 1	ECM1	Q9Z2R8_MOUSE		Y			Expressed late in development
50	Laminin, beta 2	LAMB2	LAMB2_MOUSE		Y			Stronger expression late in development

* The NIH (National Institutes of Health)/NIDCR (National Institute of Dental and Craniofacial Research) Salivary Gland Map database was accessed for this information (http://sgmap.nidcr.nih.gov/sgmap/sgexp.html). Y, positive expression.

## Supplementary Materials

Supplementary materials can be found at https://www.mdpi.com/1422-0067/21/23/9055/s1. Figure S1. Molecular network analysis of intracellular and extracellular proteins detected in the MSC and pSGC co-culture. Table S1. Putative pathways associated with secreted proteins detected in our study. Table S2. List of the relations* between internal and external differentially expressed proteins.

## Abbreviations

MSC	Mesenchymal stem cell
mMSC	mouse bone marrow-derived stem cells
GO	Gene Ontology
LC-MS/MS	Liquid chromatography tandem mass spectrometry
FN1	Fibronectin 1
AGRN	Agrin
LGALS3	Lectin, galactoside-binding, soluble, 3
CYR61	Cysteine-rich, angiogenic inducer, 61
IGFBP7	Insulin-like growth factor binding protein 7
FSTL1	Follistatin-like 1
LAMB2	Laminin, beta 2
