# Supplementary material for: Secretome Analysis of Inductive Signals for BM-MSC Transdifferentiation into Salivary Gland Progenitors
Source: Int J Mol Sci. 2020 Nov 28;21(23):9055. doi: 10.3390/ijms21239055 (PMC7730006; doi:10.3390/ijms21239055)
Supplement: Supplementary file 1 [file ijms-21-09055-s001.pdf]

Supplementary Figures and Tables

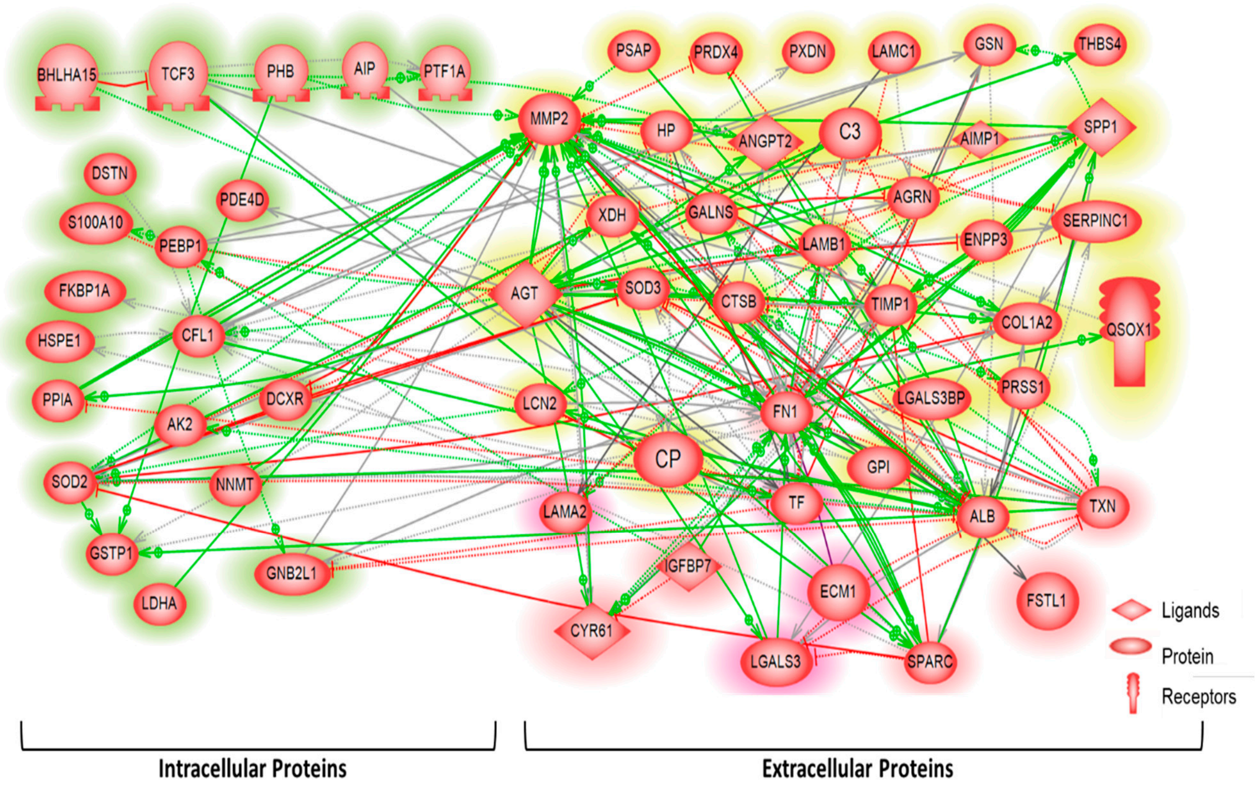

Figure S1. Molecular network analysis of intracellular and extracellular proteins detected in the MSC and pSGC co-culture. Protein interaction pathways between secreted molecules (yellow or red halos) detected in our current study and differentially expressed intracellular proteins (green halos) in the co-cultured mMSCs (green halos), which was identified by our previous ITRAQ proteomics [21], are depicted here. Among secreted molecules associated with cell differentiation (yellow halos), ten proteins (red halos) are known to be associated with salivary gland development according to the NIDCR Salivary Gland Map database. The thicker the interconnecting line is, the stronger is the association.

Table S1. Putative pathways associated with secreted proteins detected in our study

Secretome pathways	# of proteins	Fold Enrichment
PTK2 signaling	5	64.07
Cell motility	5	47.64
Laminin interactions	3	42.88
Protein phosphorylation	14	42.64
ECM proteoglycans	5	38.71
IGF regulation	15	35.96
Platelet degranulation	10	27.53
Collagen degradation	5	26.93
Response to cytosolic Ca2+	10	26.54
Signaling by MET	5	24.45
Activation of MMP	3	21.86
Collagen assembly	4	21.23
Degradation of ECM	8	20.94
Signaling by PDGF	3	19.56
Integrin cell surface interactions	4	18.82
Extracellular matrix organization	15	17.58
Platelet activation, signaling and aggregation	11	15.66
Collagen formation	4	15.17
Neutrophil degranulation	13	8.74
Hemostasis	14	8.71
Receptor Tyrosine Kinases Signaling	8	7.38
Innate Immune System	15	5.36
Metabolism of proteins	20	4.32
Post-translational protein modification	15	4.21
Immune System	18	3.78

Table S2. List of the relations between internal and external differentially expressed proteins.

RELATION	TYPE	REFERENCE
**TIMP1 ---> MMP2**	Expression	info:pmid/19000419#abs:7
**TIMP1 ---> MMP2**	Expression	info:pmid/22701711#abs:8
**TIMP1 ---> MMP2**	Expression	info:pmid/24616631#abs:6
**TIMP1 ---> MMP2**	Expression	info:pmid/22359633#abs:13
**TIMP1 ---> MMP2**	Expression	info:pmid/18292237#body:254
**TIMP1 ---> MMP2**	Expression	info:pmid/24143225#cont:188
**TIMP1 ---> MMP2**	Expression	info:pmid/15910719#body:70
**TIMP1 ---> MMP2**	Expression	info:pmid/23715597#cont:167
**TIMP1 ---> MMP2**	Expression	info:pmid/22962598#cont:235
**TIMP1 ---> MMP2**	Expression	info:pmid/18535174#body:100
**TIMP1 ---> MMP2**	Expression	info:pmid/23715826#cont:30
**TIMP1 ---> MMP2**	Expression	info:pmid/10951240#body:275
**TIMP1 ---> MMP2**	Expression	info:pmid/15808571#body:64
**TIMP1 ---> MMP2**	Expression	info:pmid/15044441#body:64
**TIMP1 ---> MMP2**	Expression	info:pmid/20130273#cont:43
**TIMP1 ---> MMP2**	Expression	info:pmid/23474083#body:140

**TIMP1 ---> MMP2**	Expression	info:doi/10.1053/j.semvascsurg.2004.12.009#body:139
**TIMP1 ---| MMP2**	DirectRegulation	info:pmid/17178348#abs:9
**TIMP1 ---| MMP2**	DirectRegulation	info:pmid/19494241#abs:6
**TIMP1 ---| MMP2**	DirectRegulation	info:pmid/16469749#abs:4
**TIMP1 ---| MMP2**	DirectRegulation	info:pmid/10613731#abs:7
**TIMP1 ---| MMP2**	DirectRegulation	info:pmid/11213361#abs:7
**TIMP1 ---| MMP2**	DirectRegulation	info:pmid/16565411#abs:5
**TIMP1 ---| MMP2**	DirectRegulation	info:pmid/1649175#abs:6
**TIMP1 ---| MMP2**	DirectRegulation	info:pmid/15301961#abs:9
**TIMP1 ---| MMP2**	DirectRegulation	info:pmid/12927692#abs:7
**TIMP1 ---| MMP2**	DirectRegulation	info:pmid/15389776#abs:10
**TIMP1 ---| MMP2**	DirectRegulation	info:pmid/1373424#abs:2
**TIMP1 ---| MMP2**	DirectRegulation	info:pmid/18661340#abs:4
**TIMP1 ---| MMP2**	DirectRegulation	info:pmid/17652746#abs:8
**TIMP1 ---| MMP2**	DirectRegulation	info:pmid/22701711#abs:9
**TIMP1 ---| MMP2**	DirectRegulation	info:pmid/8770951#abs:9
**TIMP1 ---| MMP2**	DirectRegulation	info:pmid/9006321#abs:2
**TIMP1 ---| MMP2**	DirectRegulation	info:pmid/16208432#abs:7
**TIMP1 ---| MMP2**	DirectRegulation	info:pmid/11984824#abs:15
**TIMP1 ---| MMP2**	DirectRegulation	info:pmid/24616631#abs:6
**TIMP1 ---| MMP2**	DirectRegulation	info:pmid/17878270#abs:4
**TIMP1 ---| MMP2**	DirectRegulation	info:pmid/17641224#abs:2
**TIMP1 ---| MMP2**	DirectRegulation	info:pmid/22169305#abs:9
**TIMP1 ---| MMP2**	DirectRegulation	info:pmid/23555662#cont:233
**TIMP1 ---| MMP2**	DirectRegulation	info:pmid/11278606#body:360
**TIMP1 ---| MMP2**	DirectRegulation	info:pmid/9368077#body:197
**TIMP1 ---| MMP2**	DirectRegulation	info:pmid/24445078#body:116
**TIMP1 ---| MMP2**	DirectRegulation	info:pmid/15919492#body:85
**TIMP1 ---| MMP2**	DirectRegulation	info:pmid/21782897#body:96
**TIMP1 ---| MMP2**	DirectRegulation	info:pmid/17301221#body:198
**TIMP1 ---| MMP2**	DirectRegulation	info:pmid/24093057#cont:280
**TIMP1 ---| MMP2**	DirectRegulation	info:pmid/17082773#body:181
**TIMP1 ---| MMP2**	DirectRegulation	info:pmid/12446017#body:44
**TIMP1 ---| MMP2**	DirectRegulation	info:pmid/9755855#body:126
**TIMP1 ---| MMP2**	DirectRegulation	info:pmid/21334717#body:16
**TIMP1 ---| MMP2**	DirectRegulation	info:pmid/12901881#body:14
**TIMP1 ---| MMP2**	DirectRegulation	info:pmid/19135163#body:162
**TIMP1 ---| MMP2**	DirectRegulation	info:pmid/12788377#body:33
**TIMP1 ---| MMP2**	DirectRegulation	info:pmid/14561647#body:231
**TIMP1 ---| MMP2**	DirectRegulation	info:pmid/24194630#cont:362
**TIMP1 ---| MMP2**	DirectRegulation	info:pmid/21518756#cont:291
**TIMP1 ---| MMP2**	DirectRegulation	info:pmid/19653123#cont:287
**TIMP1 ---| MMP2**	DirectRegulation	info:pmid/10366192#body:272
**TIMP1 ---| MMP2**	DirectRegulation	info:pmid/22076386#cont:33
**TIMP1 ---| MMP2**	DirectRegulation	info:pmid/10711680#body:195
**TIMP1 ---| MMP2**	DirectRegulation	info:pmid/18472160#body:13
**TIMP1 ---| MMP2**	DirectRegulation	info:pmid/19864400#body:195
**TIMP1 ---| MMP2**	DirectRegulation	info:pmid/22350743#cont:331
**TIMP1 ---| MMP2**	DirectRegulation	info:pmid/15919083#body:63
**TIMP1 ---| MMP2**	DirectRegulation	info:pmid/21946060#body:81

**TIMP1 ---| MMP2**	DirectRegulation	info:pmid/12530966#body:34
**TIMP1 ---| MMP2**	DirectRegulation	info:pmid/12620642#body:100
**TIMP1 ---| MMP2**	DirectRegulation	info:pmid/12514086#body:52
**TIMP1 ---| MMP2**	DirectRegulation	info:pmid/19933182#cont:180
**TIMP1 ---| MMP2**	DirectRegulation	info:pmid/17533011#body:17
**TIMP1 ---| MMP2**	DirectRegulation	info:pmid/19046765#body:143
**TIMP1 ---| MMP2**	DirectRegulation	info:pmid/9846979#body:140
**TIMP1 ---| MMP2**	DirectRegulation	info:pmid/22100390#cont:65
**TIMP1 ---| MMP2**	DirectRegulation	info:pmid/21145716#body:23
**TIMP1 ---| MMP2**	DirectRegulation	info:pmid/15950241#body:137
**TIMP1 ---| MMP2**	DirectRegulation	info:pmid/19944523#body:91
**TIMP1 ---| MMP2**	DirectRegulation	info:pmid/16603142#body:6
**TIMP1 ---| MMP2**	DirectRegulation	info:pmid/21537327#cont:25
**TIMP1 ---| MMP2**	DirectRegulation	info:pmid/18375305#body:144
**TIMP1 ---| MMP2**	DirectRegulation	info:pmid/23228413#body:12
**TIMP1 ---| MMP2**	DirectRegulation	info:pmid/18602101#body:136
**TIMP1 ---| MMP2**	DirectRegulation	info:pmid/9716492#body:205
**TIMP1 ---| MMP2**	DirectRegulation	info:pmid/10559137#body:84
**TIMP1 ---| MMP2**	DirectRegulation	info:pmid/9570393#body:69
**TIMP1 ---| MMP2**	DirectRegulation	info:pmid/10405207#body:52
**TIMP1 ---| MMP2**	DirectRegulation	info:pmid/12542540#body:61
**TIMP1 ---| MMP2**	DirectRegulation	info:pmid/16488444#body:165
**TIMP1 ---| MMP2**	DirectRegulation	info:pmid/23878609#cont:979
**TIMP1 ---| MMP2**	DirectRegulation	info:pmid/17412577#body:82
**TIMP1 ---| MMP2**	DirectRegulation	info:pmid/17822657#body:30
**TIMP1 ---| MMP2**	DirectRegulation	info:pmid/20145075#body:158
**TIMP1 ---| MMP2**	DirectRegulation	info:pmid/18215140#body:194
**TIMP1 ---| MMP2**	DirectRegulation	info:pmid/22030200#body:78
**TIMP1 ---| MMP2**	DirectRegulation	info:pmid/16770011#body:82
**TIMP1 ---| MMP2**	DirectRegulation	info:pmid/16487958#body:132
**TIMP1 ---| MMP2**	DirectRegulation	info:pmid/21731773#cont:19
**TIMP1 ---| MMP2**	DirectRegulation	info:pmid/23428182#body:111
**TIMP1 ---| MMP2**	DirectRegulation	info:pmid/19136391#body:283
**TIMP1 ---| MMP2**	DirectRegulation	info:pmid/19523695#body:43
**TIMP1 ---| MMP2**	DirectRegulation	info:pmid/11319136#body:237
**TIMP1 ---| MMP2**	DirectRegulation	info:pmid/17702617#body:17
**TIMP1 ---| MMP2**	DirectRegulation	info:pmid/12374789#body:328
**TIMP1 ---| MMP2**	DirectRegulation	info:pmid/10081908#body:7
**TIMP1 ---| MMP2**	DirectRegulation	info:pmid/18312928#body:175
**TIMP1 ---| MMP2**	DirectRegulation	info:pmid/15313474#body:175
**TIMP1 ---| MMP2**	DirectRegulation	info:pmid/20837483#cont:37
**TIMP1 ---| MMP2**	DirectRegulation	info:pmid/23113307#cont:20
**TIMP1 ---| MMP2**	DirectRegulation	info:pmid/16112109#body:121
**TIMP1 ---| MMP2**	DirectRegulation	info:pmid/12852489#body:303
**TIMP1 ---| MMP2**	DirectRegulation	info:pmid/19157825#body:122
**TIMP1 ---| MMP2**	DirectRegulation	info:pmid/10996723#body:8
**TIMP1 ---| MMP2**	DirectRegulation	info:pmid/11751597#body:217
**TIMP1 ---| MMP2**	DirectRegulation	info:pmid/20123196#body:129
**TIMP1 ---| MMP2**	DirectRegulation	info:pmid/20683769#cont:34
**TIMP1 ---| MMP2**	DirectRegulation	info:pmid/21409414#cont:23

**TIMP1 ---| MMP2**	DirectRegulation	info:pmid/10366632#body:271
**AGT --+> MMP2**	Expression	info:pmid/19082717#abs:2
**AGT --+> MMP2**	Expression	info:pmid/22590832#abs:8
**AGT --+> MMP2**	Expression	info:pmid/22340635#abs:6
**AGT --+> MMP2**	Expression	info:pmid/24416421#abs:6
**AGT --+> MMP2**	Expression	info:pmid/23006733#abs:2
**AGT --+> MMP2**	Expression	info:pmid/15522203#abs:5
**AGT --+> MMP2**	Expression	info:pmid/10512375#abs:13
**AGT --+> MMP2**	Expression	info:pmid/16728425#abs:12
**AGT --+> MMP2**	Expression	info:pmid/21952436#abs:6
**AGT --+> MMP2**	Expression	info:pmid/19581512#abs:8
**AGT --+> MMP2**	Expression	info:pmid/16713603#abs:7
**AGT --+> MMP2**	Expression	info:pmid/22878980#abs:3
**AGT --+> MMP2**	Expression	info:pmid/18296491#abs:5
**AGT --+> MMP2**	Expression	info:pmid/11454555#abs:6
**AGT --+> MMP2**	Expression	info:pmid/22561688#abs:6
**AGT --+> MMP2**	Expression	info:pmid/20940000#abs:6
**AGT --+> MMP2**	Expression	info:pmid/23676189#abs:13
**AGT --+> MMP2**	Expression	info:pmid/23841699#abs:6
**AGT --+> MMP2**	Expression	info:pmid/24095877#body:164
**AGT --+> MMP2**	Expression	info:pmid/21769437#cont:119
**AGT --+> MMP2**	Expression	info:pmid/24447911#body:111
**AGT --+> MMP2**	Expression	info:pmid/24219285#cont:215
**AGT --+> MMP2**	Expression	info:pmid/23127783#body:180
**AGT --+> MMP2**	Expression	info:pmid/16857167#body:130
**AGT --+> MMP2**	Expression	info:pmid/22519443#cont:177
**AGT --+> MMP2**	Expression	info:pmid/21185709#body:17
**AGT --+> MMP2**	Expression	info:pmid/23667404#body:74
**AGT --+> MMP2**	Expression	info:pmid/23041273#body:187
**AGT --+> MMP2**	Expression	info:pmid/24358274#cont:155
**AGT --+> MMP2**	Expression	info:pmid/19460403#body:283
**AGT --+> MMP2**	Expression	info:pmid/23316965#cont:39
**AGT --+> MMP2**	Expression	info:pmid/22982961#body:146
**AGT --+> MMP2**	Expression	info:pmid/15350851#body:94
**AGT --+> MMP2**	Expression	info:pmid/16251426#body:176
**AGT --+> MMP2**	Expression	info:pmid/19281810#body:211
**AGT --+> MMP2**	Expression	info:pmid/15670768#body:59
**AGT --+> MMP2**	Expression	info:pmid/21464389#cont:247
**AGT --+> MMP2**	Expression	info:pmid/21719784#cont:189
**AGT --+> MMP2**	Expression	info:pmid/17717147#body:96
**AGT --+> MMP2**	Expression	info:pmid/12371906#body:61
**AGT --+> MMP2**	Expression	info:pmid/21245762#cont:173
**AGT --+> MMP2**	Expression	info:pmid/14962948#body:162
**AGT --+> MMP2**	Expression	info:pmid/22100792#body:577
**AGT --+> MMP2**	Expression	info:pmid/21881526#cont:187
**AGT --+> MMP2**	Expression	info:pmid/18048061#body:112
**AGT --+> MMP2**	Expression	info:pmid/24393555#cont:137
**AGT --+> MMP2**	Expression	info:pmid/16166267#body:245
**AGT --+> MMP2**	Expression	info:pmid/20409863#body:352
**AGT --+> MMP2**	Expression	info:pmid/17452499#body:157

**AGT --+> MMP2**	Expression	info:pmid/21718678#body:145
**AGT --+> MMP2**	Expression	info:pmid/22813854#body:98
**AGT --+> MMP2**	Expression	info:pmid/24161906#cont:164
**AGT --+> MMP2**	Expression	info:pmid/22083158#cont:570
**AGT --+> MMP2**	Expression	info:pmid/23073243#body:119
**AGT --+> MMP2**	Expression	info:pmid/20184869#body:50
**AGT --+> MMP2**	Expression	info:pmid/23446230#cont:132
**AGT --+> MMP2**	Expression	info:doi/10.1016/j.yjmcc.2006.04.019#body:94
**AGT --+> MMP2**	Expression	info:doi/10.1016/j.vph.2006.08.210#body:9
**AGT --+> MMP2**	Expression	info:doi/10.1016/j.ddmod.2011.02.001#body:82
**AGT --+> MMP2**	Expression	info:doi/10.1016/j.ijcard.2012.03.137#body:3
**AGT --+> MMP2**	Expression	info:doi/10.1016/j.ajpath.2011.02.006#body:232
**AGT --+> MMP2**	MolTransport	info:pmid/18619957#abs:4
**AGT --+> MMP2**	MolTransport	info:pmid/14644777#abs:6
**AGT --+> MMP2**	MolTransport	info:pmid/11602818#abs:5
**AGT --+> MMP2**	MolTransport	info:pmid/19729613#body:192
**AGT --+> MMP2**	MolTransport	info:pmid/17336987#body:142
**AGT --+> MMP2**	MolTransport	info:pmid/22982961#body:96
**AGT --+> MMP2**	MolTransport	info:pmid/19460403#body:154
**AGT --+> MMP2**	MolTransport	info:pmid/21271181#cont:164
**AGT --+> MMP2**	MolTransport	info:pmid/15670768#body:83
**AGT --+> MMP2**	MolTransport	info:pmid/20606107#cont:264
**AGT --+> MMP2**	MolTransport	info:pmid/22519445#cont:585
**AGT --+> MMP2**	MolTransport	info:pmid/20821936#cont:187
**AGT --+> MMP2**	MolTransport	info:pmid/19094982#body:146
**AGT --+> MMP2**	MolTransport	info:pmid/21920892#cont:410
**AGT --+> MMP2**	MolTransport	info:pmid/16864744#body:67
**AGT --+> XDH**	Expression	info:pmid/11862018#abs:5
**AGT --+> XDH**	Expression	info:pmid/22429089#cont:215
**AGT --+> XDH**	Expression	info:pmid/21644836#cont:183
**AGT --+> XDH**	Expression	info:pmid/17234726#title:1
**AGT --+> XDH**	Expression	info:pmid/17377148#body:59
**FN1 --+> MMP2**	Expression	info:pmid/19590887#abs:12
**FN1 --+> MMP2**	Expression	info:pmid/18540849#abs:7
**FN1 --+> MMP2**	Expression	info:pmid/10515879#abs:5
**FN1 --+> MMP2**	Expression	info:pmid/17341207#abs:6
**FN1 --+> MMP2**	Expression	info:pmid/16788844#abs:6
**FN1 --+> MMP2**	Expression	info:pmid/11776634#abs:11
**FN1 --+> MMP2**	Expression	info:pmid/17255325#abs:5
**FN1 --+> MMP2**	Expression	info:pmid/7789549#abs:11
**FN1 --+> MMP2**	Expression	info:pmid/15583082#body:242
**FN1 --+> MMP2**	Expression	info:pmid/22292748#cont:64
**FN1 --+> MMP2**	Expression	info:pmid/22683701#body:198
**FN1 --+> MMP2**	Expression	info:pmid/23826082#cont:326
**FN1 --+> MMP2**	Expression	info:pmid/18162175#body:101
**FN1 --+> MMP2**	Expression	info:pmid/18243246#body:25
**FN1 --+> MMP2**	Expression	info:pmid/23895273#cont:226
**FN1 --+> MMP2**	Expression	info:pmid/23683757#body:162
**FN1 --+> MMP2**	Expression	info:pmid/15588763#body:350
**FN1 --+> MMP2**	Expression	info:pmid/23563505#cont:262

**FN1 --+> MMP2**	Expression	info:pmid/21843641#body:280
**FN1 --+> MMP2**	Expression	info:pmid/20159608#body:180
**FN1 --+> MMP2**	Expression	info:pmid/15120641#body:126
**FN1 --+> MMP2**	Expression	info:pmid/19105967#body:173
**FN1 --+> MMP2**	Expression	info:pmid/11495705#body:161
**FN1 --+> MMP2**	Expression	info:pmid/22354151#cont:230
**FN1 --+> MMP2**	Expression	info:pmid/21147512#body:156
**FN1 --+> MMP2**	Expression	info:pmid/17707437#body:102
**FN1 --+> MMP2**	Expression	info:pmid/23935989#cont:268
**FN1 --+> MMP2**	Expression	info:pmid/20425121#cont:161
**FN1 --+> MMP2**	Expression	info:pmid/23261429#body:47
**FN1 --+> MMP2**	Expression	info:pmid/15967869#body:148
**FN1 --+> MMP2**	Expression	info:doi/10.2353/ajpath.2010.090200#body:180
**FN1 --+> MMP2**	Expression	info:doi/10.1016/j.yjmcc.2011.12.004#body:129
**FN1 --+> MMP2**	Expression	info:doi/10.1016/S0002-9440(10)61131-5#body:105
**FN1 --+> MMP2**	Expression	info:doi/10.1016/j.fertnstert.2006.07.935#body:18
**FN1 --+> MMP2**	Expression	info:doi/10.2353/ajpath.2007.060456#body:112
**MMP2 ---| FN1**	Expression	info:pmid/21115719#abs:10
**MMP2 ---| FN1**	Expression	info:pmid/22696147#cont:260
**MMP2 ---| FN1**	Expression	info:pmid/23201304#title:1
**MMP2 ---| FN1**	Expression	info:pmid/15734687#body:184
**MMP2 ---| FN1**	Expression	info:pmid/14764809#body:284
**MMP2 ---| FN1**	Expression	info:pmid/21750048#cont:195
**MMP2 ---| FN1**	Expression	info:pmid/17604018#body:150
**MMP2 ---| FN1**	Expression	info:pmid/19276073#body:163
**MMP2 ---| FN1**	Expression	info:pmid/20409863#body:341
**MMP2 ---| FN1**	Expression	info:pmid/9916016#body:219
**MMP2 ---| FN1**	Expression	info:pmid/22100792#body:593
**MMP2 ---| FN1**	Expression	info:pmid/21881526#cont:223
**MMP2 ---| FN1**	Expression	info:pmid/12855272#body:142
**MMP2 ---| FN1**	Expression	info:pmid/16732041#body:99
**MMP2 ---| FN1**	Expression	info:embase/2011567940#cont:73
**FN1 --+> MMP2**	MolTransport	info:pmid/16603966#abs:8
**FN1 --+> MMP2**	MolTransport	info:pmid/11776634#abs:5
**FN1 --+> MMP2**	MolTransport	info:pmid/15120641#body:101
**FN1 --+> MMP2**	MolTransport	info:pmid/11782389#title:1
**FN1 --+> MMP2**	MolTransport	info:pmid/18028052#title:1
**FN1 --+> MMP2**	MolTransport	info:pmid/22534113#body:130
**FN1 --+> MMP2**	MolTransport	info:pmid/11495705#body:159
**FN1 --+> MMP2**	MolTransport	info:pmid/22594107#cont:272
**FN1 --+> MMP2**	MolTransport	info:pmid/11134254#body:209
**FN1 --+> MMP2**	MolTransport	info:pmid/11953835#body:164
**FN1 --+> MMP2**	MolTransport	info:doi/10.1016/j.cyto.2012.03.025#body:130
**LGALS3 --+> FN1**	DirectRegulation	info:pmid/23118221#abs:8
**LGALS3 --+> FN1**	DirectRegulation	info:pmid/8621934#abs:6
**LGALS3 --+> FN1**	DirectRegulation	info:pmid/16581792#abs:7
**LGALS3 --+> FN1**	DirectRegulation	info:pmid/20363921#body:64
**LGALS3 --+> FN1**	DirectRegulation	info:pmid/18272359#body:126
**LGALS3 --+> FN1**	DirectRegulation	info:pmid/14579602#body:114
**LGALS3 --+> FN1**	DirectRegulation	info:pmid/22527325#cont:41

**LGALS3 --+> FN1**	DirectRegulation	info:pmid/21094672#body:127
**LGALS3 --+> FN1**	DirectRegulation	info:pmid/15181153#body:253
**LGALS3 --+> FN1**	DirectRegulation	info:pmid/18347068#body:114
**LGALS3 --+> FN1**	DirectRegulation	info:pmid/18806266#body:309
**LGALS3 --+> FN1**	DirectRegulation	info:pmid/16837064#body:24
**LGALS3 --+> FN1**	DirectRegulation	info:pmid/21616652#body:54
**LGALS3 --+> FN1**	DirectRegulation	info:pmid/19940114#body:381
**LGALS3 --+> FN1**	DirectRegulation	info:pmid/12072370#body:78
**LGALS3 --+> FN1**	DirectRegulation	info:pmid/15800063#body:64
**LGALS3 --+> FN1**	DirectRegulation	info:pmid/12767519#body:94
**LGALS3 --+> FN1**	DirectRegulation	info:pmid/23657817#cont:782
**LGALS3 --+> FN1**	DirectRegulation	info:pmid/22465244#body:4
**LGALS3 --+> FN1**	DirectRegulation	info:doi/10.1016/j.neulet.2012.02.065#body:4
**CP --+> TF**	DirectRegulation	info:pmid/8828436#abs:1
**CP --+> TF**	DirectRegulation	info:pmid/19005224#abs:2
**CP --+> TF**	DirectRegulation	info:pmid/16629165#abs:5
**CP --+> TF**	DirectRegulation	info:pmid/17919118#body:131
**CP --+> TF**	DirectRegulation	info:pmid/17651808#body:72
**CP --+> TF**	DirectRegulation	info:pmid/19783267#body:99
**CP --+> TF**	DirectRegulation	info:pmid/23815406#cont:255
**CP --+> TF**	DirectRegulation	info:pmid/12430618#body:259
**CP --+> TF**	DirectRegulation	info:pmid/22535765#cont:354
**CP --+> TF**	DirectRegulation	info:pmid/17921041#body:159
**CP --+> TF**	DirectRegulation	info:pmid/9873059#body:163
**CP --+> TF**	DirectRegulation	info:pmid/19717781#body:197
**CP --+> TF**	DirectRegulation	info:pmid/9584616#body:33
**CP --+> TF**	DirectRegulation	info:pmid/10882071#body:20
**CP --+> TF**	DirectRegulation	info:pmid/22040722#body:116
**CP --+> TF**	DirectRegulation	info:pmid/18187051#body:85
**CP --+> TF**	DirectRegulation	info:pmid/24388876#body:211
**CP --+> TF**	DirectRegulation	info:pmid/24036104#body:104
**CP --+> TF**	DirectRegulation	info:pmid/17227764#body:263
**CP --+> TF**	DirectRegulation	info:pmid/16678014#body:106
**CP --+> TF**	DirectRegulation	info:pmid/10491642#body:256
**CP --+> TF**	DirectRegulation	info:pmid/20386880#cont:53
**CP --+> TF**	DirectRegulation	info:pmid/17101452#body:48
**CP --+> TF**	DirectRegulation	info:pmid/16778276#body:148
**CP --+> TF**	DirectRegulation	info:pmid/15623795#body:228
**CP --+> TF**	DirectRegulation	info:doi/10.1053/ob.1996.v175.a74252#body:97
**CP ---> TF**	MolTransport	info:pmid/1992468#abs:7
**CP ---> TF**	MolTransport	info:pmid/17227764#body:313
**CP ---> TF**	MolTransport	info:pmid/12356832#body:283
**CP ---> TF**	MolTransport	info:doi/10.1016/j.foodchem.2008.06.012#body:3
**SPARC --+> MMP2**	Expression	info:pmid/17490812#abs:2
**SPARC --+> MMP2**	Expression	info:pmid/12201246#abs:11
**SPARC --+> MMP2**	Expression	info:pmid/17088972#cont:229
**SPARC --+> MMP2**	Expression	info:pmid/22120277#body:180
**SPARC --+> MMP2**	Expression	info:pmid/22879971#cont:551
**SPARC --+> MMP2**	Expression	info:pmid/16084059#body:11
**SPARC --+> MMP2**	Expression	info:pmid/18849185#body:185

**SPARC --+> MMP2**	Expression	info:pmid/23516489#cont:632
**SPARC --+> MMP2**	Expression	info:pmid/10571408#body:7
**SPARC --+> MMP2**	Expression	info:pmid/21384171#cont:43
**SPARC --+> MMP2**	Expression	info:doi/10.1016/j.wneu.2011.09.005#body:180
**AGT --+> SPP1**	Expression	info:pmid/12620700#abs:2
**AGT --+> SPP1**	Expression	info:pmid/18712054#abs:8
**AGT --+> SPP1**	Expression	info:pmid/21304262#abs:12
**AGT --+> SPP1**	Expression	info:pmid/10807582#abs:8
**AGT --+> SPP1**	Expression	info:pmid/11382929#abs:7
**AGT --+> SPP1**	Expression	info:pmid/8941637#abs:5
**AGT --+> SPP1**	Expression	info:pmid/19327775#abs:11
**AGT --+> SPP1**	Expression	info:pmid/18785976#abs:13
**AGT --+> SPP1**	Expression	info:pmid/18156195#abs:7
**AGT --+> SPP1**	Expression	info:pmid/14755545#abs:15
**AGT --+> SPP1**	Expression	info:pmid/9844126#abs:6
**AGT --+> SPP1**	Expression	info:pmid/16330679#abs:5
**AGT --+> SPP1**	Expression	info:pmid/23466073#abs:5
**AGT --+> SPP1**	Expression	info:pmid/19000373#abs:9
**AGT --+> SPP1**	Expression	info:pmid/14978165#abs:3
**AGT --+> SPP1**	Expression	info:pmid/15997171#abs:10
**AGT --+> SPP1**	Expression	info:pmid/8924518#abs:7
**AGT --+> SPP1**	Expression	info:pmid/19080183#abs:12
**AGT --+> SPP1**	Expression	info:pmid/22822546#abs:5
**AGT --+> SPP1**	Expression	info:pmid/20130530#abs:7
**AGT --+> SPP1**	Expression	info:pmid/18511847#abs:6
**AGT --+> SPP1**	Expression	info:pmid/15121739#abs:5
**AGT --+> SPP1**	Expression	info:pmid/16027079#abs:11
**AGT --+> SPP1**	Expression	info:pmid/15387967#abs:1
**AGT --+> SPP1**	Expression	info:pmid/19225557#abs:6
**AGT --+> SPP1**	Expression	info:pmid/15576638#abs:8
**AGT --+> SPP1**	Expression	info:pmid/10828755#abs:2
**AGT --+> SPP1**	Expression	info:pmid/16528250#abs:8
**AGT --+> SPP1**	Expression	info:pmid/11012882#abs:2
**AGT --+> SPP1**	Expression	info:pmid/10611911#abs:7
**AGT --+> SPP1**	Expression	info:pmid/14987565#body:152
**AGT --+> SPP1**	Expression	info:pmid/10642310#body:111
**AGT --+> SPP1**	Expression	info:pmid/21949681#cont:49
**AGT --+> SPP1**	Expression	info:pmid/14597759#body:66
**AGT --+> SPP1**	Expression	info:pmid/15933246#body:145
**AGT --+> SPP1**	Expression	info:pmid/20031444#body:96
**AGT --+> SPP1**	Expression	info:pmid/18037370#body:104
**AGT --+> SPP1**	Expression	info:pmid/12970113#body:202
**AGT --+> SPP1**	Expression	info:pmid/20127409#cont:83
**AGT --+> SPP1**	Expression	info:pmid/12676174#body:108
**AGT --+> SPP1**	Expression	info:pmid/15123578#body:147
**AGT --+> SPP1**	Expression	info:pmid/11979338#body:134
**AGT --+> SPP1**	Expression	info:pmid/19801149#body:70
**AGT --+> SPP1**	Expression	info:pmid/20664555#cont:138
**AGT --+> SPP1**	Expression	info:pmid/12089373#body:306
**AGT --+> SPP1**	Expression	info:pmid/8952596#body:125

**AGT --+> SPP1**	Expression	info:pmid/24247243#cont:298
**AGT --+> SPP1**	Expression	info:pmid/19573532#body:84
**AGT --+> SPP1**	Expression	info:pmid/10432396#body:203
**AGT --+> SPP1**	Expression	info:pmid/17218415#body:138
**AGT --+> SPP1**	Expression	info:pmid/16520260#body:75
**AGT --+> SPP1**	Expression	info:pmid/19106220#body:60
**AGT --+> SPP1**	Expression	info:pmid/12945227#title:1
**AGT --+> SPP1**	Expression	info:pmid/11012898#body:48
**AGT --+> SPP1**	Expression	info:pmid/12522128#body:155
**AGT --+> SPP1**	Expression	info:pmid/22036853#body:23
**AGT --+> SPP1**	Expression	info:pmid/15978615#body:85
**AGT --+> SPP1**	Expression	info:pmid/12414515#body:4
**AGT --+> SPP1**	Expression	info:pmid/9561998#body:73
**AGT --+> SPP1**	Expression	info:pmid/23334241#cont:235
**AGT --+> SPP1**	Expression	info:pmid/21223972#body:91
**AGT --+> SPP1**	Expression	info:pmid/12826287#body:125
**AGT --+> SPP1**	Expression	info:pmid/22210452#body:141
**AGT --+> SPP1**	Expression	info:pmid/15826947#body:335
**AGT --+> SPP1**	Expression	info:pmid/15120833#body:83
**AGT --+> SPP1**	Expression	info:pmid/12060392#body:466
**AGT --+> SPP1**	Expression	info:pmid/9386176#body:238
**AGT --+> SPP1**	Expression	info:pmid/10977781#body:67
**AGT --+> SPP1**	Expression	info:pmid/11318933#body:171
**AGT --+> SPP1**	Expression	info:pmid/22283779#cont:155
**AGT --+> SPP1**	Expression	info:pmid/18579222#body:32
**AGT --+> SPP1**	Expression	info:pmid/11115077#body:262
**AGT --+> SPP1**	Expression	info:pmid/11891192#body:139
**AGT --+> SPP1**	Expression	info:pmid/21034455#cont:170
**AGT --+> SPP1**	Expression	info:pmid/17413042#body:244
**AGT --+> SPP1**	Expression	info:pmid/15557322#body:208
**AGT --+> SPP1**	Expression	info:pmid/8911275#body:193
**AGT --+> SPP1**	Expression	info:pmid/24315461#body:75
**AGT --+> SPP1**	Expression	info:pmid/21495969#cont:209
**AGT --+> SPP1**	Expression	info:pmid/18638447#body:74
**AGT --+> SPP1**	Expression	info:pmid/10024324#body:43
**AGT --+> SPP1**	Expression	info:pmid/20447565#body:91
**AGT --+> SPP1**	Expression	info:pmid/7583583#body:203
**AGT --+> SPP1**	Expression	info:pmid/24183793#body:85
**AGT --+> SPP1**	Expression	info:pmid/17301319#body:81
**AGT --+> SPP1**	Expression	info:pmid/18645046#body:198
**AGT --+> SPP1**	Expression	info:pmid/12920127#body:285
**AGT --+> SPP1**	Expression	info:pmid/19002105#body:215
**AGT --+> SPP1**	Expression	info:pmid/12756220#body:168
**AGT --+> SPP1**	Expression	info:pmid/15117918#body:44
**AGT --+> SPP1**	Expression	info:pmid/9012655#body:36
**AGT --+> SPP1**	Expression	info:pmid/15520299#body:48
**AGT --+> SPP1**	Expression	info:doi/10.1016/S0025-7125(03)00125-1#body:77
**AGT --+> SPP1**	Expression	info:doi/10.1016/S0270-9295(03)00090-1#body:157
**AGT --+> SPP1**	Expression	info:doi/10.2174/1381612811319170007#cont:266
**AGT --+> SPP1**	Expression	info:doi/10.1016/j.cardfail.2006.08.090#body:7

**AGT --+> SPP1**	Expression	info:doi/10.1016/j.fitote.2012.02.010#body:163
**AGT --+> SPP1**	Expression	info:doi/10.1016/S0272-6386(00)70031-X#body:12
**AGT --+> SPP1**	Expression	info:doi/10.1016/j.lfs.2003.08.036#body:124
**LGALS3 --+> MMP2**	Expression	info:pmid/21448903#abs:9
**LGALS3 --+> MMP2**	Expression	info:pmid/18988806#body:259
**LGALS3 --+> MMP2**	Expression	info:pmid/24530298#body:71
**AGT --+> FN1**	Expression	info:pmid/1848253#abs:7
**AGT --+> FN1**	Expression	info:pmid/2254339#abs:6
**AGT --+> FN1**	Expression	info:pmid/9756884#abs:2
**AGT --+> FN1**	Expression	info:pmid/23535586#abs:11
**AGT --+> FN1**	Expression	info:pmid/11737589#abs:1
**AGT --+> FN1**	Expression	info:pmid/9407495#abs:6
**AGT --+> FN1**	Expression	info:pmid/16987006#abs:4
**AGT --+> FN1**	Expression	info:pmid/21223972#abs:9
**AGT --+> FN1**	Expression	info:pmid/12801513#abs:4
**AGT --+> FN1**	Expression	info:pmid/20662702#abs:9
**AGT --+> FN1**	Expression	info:pmid/11786549#abs:6
**AGT --+> FN1**	Expression	info:pmid/15493478#abs:4
**AGT --+> FN1**	Expression	info:pmid/12372774#abs:7
**AGT --+> FN1**	Expression	info:pmid/22357921#abs:3
**AGT --+> FN1**	Expression	info:pmid/9403620#abs:9
**AGT --+> FN1**	Expression	info:pmid/8282345#abs:9
**AGT --+> FN1**	Expression	info:pmid/8206611#abs:6
**AGT --+> FN1**	Expression	info:pmid/7755479#abs:13
**AGT --+> FN1**	Expression	info:pmid/11108151#abs:2
**AGT --+> FN1**	Expression	info:pmid/20490773#abs:7
**AGT --+> FN1**	Expression	info:pmid/8518542#abs:8
**AGT --+> FN1**	Expression	info:pmid/18756259#abs:7
**AGT --+> FN1**	Expression	info:pmid/10325245#abs:4
**AGT --+> FN1**	Expression	info:pmid/7858853#abs:6
**AGT --+> FN1**	Expression	info:pmid/15770925#abs:8
**AGT --+> FN1**	Expression	info:pmid/21613793#abs:6
**AGT --+> FN1**	Expression	info:pmid/8648265#abs:8
**AGT --+> FN1**	Expression	info:pmid/21693104#abs:8
**AGT --+> FN1**	Expression	info:pmid/20679547#abs:5
**AGT --+> FN1**	Expression	info:pmid/14599716#abs:7
**AGT --+> FN1**	Expression	info:pmid/15698454#abs:12
**AGT --+> FN1**	Expression	info:pmid/20438519#abs:3
**AGT --+> FN1**	Expression	info:pmid/8137508#abs:12
**AGT --+> FN1**	Expression	info:pmid/21646597#abs:7
**AGT --+> FN1**	Expression	info:pmid/11551879#abs:7
**AGT --+> FN1**	Expression	info:pmid/14571285#abs:2
**AGT --+> FN1**	Expression	info:pmid/10389125#abs:7
**AGT --+> FN1**	Expression	info:pmid/20112290#abs:4
**AGT --+> FN1**	Expression	info:pmid/22982961#abs:7
**AGT --+> FN1**	Expression	info:pmid/8476421#abs:3
**AGT --+> FN1**	Expression	info:pmid/16720735#abs:4
**AGT --+> FN1**	Expression	info:pmid/21572015#abs:8
**AGT --+> FN1**	Expression	info:pmid/18278065#abs:5
**AGT --+> FN1**	Expression	info:pmid/8613264#abs:8

**AGT --+> FN1**	Expression	info:pmid/15576638#abs:8
**AGT --+> FN1**	Expression	info:pmid/8764262#abs:11
**AGT --+> FN1**	Expression	info:pmid/16911918#abs:6
**AGT --+> FN1**	Expression	info:pmid/18559349#body:362
**AGT --+> FN1**	Expression	info:pmid/23940049#cont:491
**AGT --+> FN1**	Expression	info:pmid/12952842#body:158
**AGT --+> FN1**	Expression	info:pmid/17592071#body:159
**AGT --+> FN1**	Expression	info:pmid/21215756#body:96
**AGT --+> FN1**	Expression	info:pmid/8996227#body:148
**AGT --+> FN1**	Expression	info:pmid/8996229#body:148
**AGT --+> FN1**	Expression	info:pmid/11230363#body:156
**AGT --+> FN1**	Expression	info:pmid/15883213#body:194
**AGT --+> FN1**	Expression	info:pmid/23875251#cont:54
**AGT --+> FN1**	Expression	info:pmid/15677310#body:134
**AGT --+> FN1**	Expression	info:pmid/11033054#body:188
**AGT --+> FN1**	Expression	info:pmid/24157579#body:79
**AGT --+> FN1**	Expression	info:pmid/14578193#body:185
**AGT --+> FN1**	Expression	info:pmid/23472618#cont:439
**AGT --+> FN1**	Expression	info:pmid/24275770#cont:472
**AGT --+> FN1**	Expression	info:pmid/19342448#body:213
**AGT --+> FN1**	Expression	info:pmid/22964022#body:100
**AGT --+> FN1**	Expression	info:pmid/19460403#body:567
**AGT --+> FN1**	Expression	info:pmid/11729234#body:268
**AGT --+> FN1**	Expression	info:pmid/12034716#body:335
**AGT --+> FN1**	Expression	info:pmid/12208585#body:49
**AGT --+> FN1**	Expression	info:pmid/18292201#body:70
**AGT --+> FN1**	Expression	info:pmid/20596034#cont:317
**AGT --+> FN1**	Expression	info:pmid/19375729#body:15
**AGT --+> FN1**	Expression	info:pmid/10715259#body:183
**AGT --+> FN1**	Expression	info:pmid/22639821#cont:87
**AGT --+> FN1**	Expression	info:pmid/8925571#title:1
**AGT --+> FN1**	Expression	info:pmid/11473637#body:233
**AGT --+> FN1**	Expression	info:pmid/22280423#cont:242
**AGT --+> FN1**	Expression	info:pmid/12707289#body:129
**AGT --+> FN1**	Expression	info:pmid/12681244#body:79
**AGT --+> FN1**	Expression	info:pmid/8127007#title:1
**AGT --+> FN1**	Expression	info:pmid/18235084#body:148
**AGT --+> FN1**	Expression	info:pmid/15576842#body:294
**AGT --+> FN1**	Expression	info:pmid/21610512#cont:239
**AGT --+> FN1**	Expression	info:pmid/17481939#body:102
**AGT --+> FN1**	Expression	info:pmid/17035613#body:212
**AGT --+> FN1**	Expression	info:pmid/17074304#body:95
**AGT --+> FN1**	Expression	info:pmid/16632126#body:119
**AGT --+> FN1**	Expression	info:pmid/10930408#body:305
**AGT --+> FN1**	Expression	info:pmid/24210651#body:6
**AGT --+> FN1**	Expression	info:pmid/15615821#body:302
**AGT --+> FN1**	Expression	info:pmid/21586275#body:58
**AGT --+> FN1**	Expression	info:pmid/10977869#body:582
**AGT --+> FN1**	Expression	info:pmid/10699153#body:393
**AGT --+> FN1**	Expression	info:pmid/19706694#cont:33

**AGT --+> FN1**	Expression	info:pmid/11566906#body:127
**AGT --+> FN1**	Expression	info:pmid/8941637#body:335
**AGT --+> FN1**	Expression	info:pmid/12761244#body:56
**AGT --+> FN1**	Expression	info:pmid/22883438#body:90
**AGT --+> FN1**	Expression	info:pmid/11696450#body:145
**AGT --+> FN1**	Expression	info:pmid/12538841#body:152
**AGT --+> FN1**	MolTransport	info:pmid/11600408#abs:4
**AGT --+> FN1**	MolTransport	info:pmid/15698454#abs:10
**AGT --+> FN1**	MolTransport	info:pmid/11737589#body:177
**AGT --+> FN1**	MolTransport	info:pmid/21152444#body:256
**AGT --+> FN1**	MolTransport	info:pmid/10325245#body:196
**AGT --+> FN1**	MolTransport	info:pmid/8996227#body:39
**AGT --+> FN1**	MolTransport	info:pmid/8996229#body:39
**AGT --+> FN1**	MolTransport	info:pmid/14578193#body:147
**AGT --+> FN1**	MolTransport	info:pmid/21881526#cont:263
**AGT --+> FN1**	MolTransport	info:pmid/16394111#body:241
**AGT --+> FN1**	MolTransport	info:pmid/11282364#body:112
**AGT --+> FN1**	MolTransport	info:pmid/15509535#body:144
**AGT --+> FN1**	MolTransport	info:pmid/17692338#body:251
**ANGPT2 --+> MMP2**	Expression	info:pmid/18182823#abs:14
**ANGPT2 --+> MMP2**	Expression	info:pmid/16424009#abs:3
**ANGPT2 --+> MMP2**	Expression	info:pmid/20950259#cont:175
**ANGPT2 --+> MMP2**	Expression	info:pmid/12861074#body:54
**ANGPT2 --+> MMP2**	Expression	info:pmid/12876214#body:212
**ANGPT2 --+> MMP2**	Expression	info:pmid/22483377#body:108
**ANGPT2 --+> MMP2**	Expression	info:doi/10.1016/j.cyto.2012.03.007#body:108
**TXN --+> MMP2**	Regulation	info:pmid/14871484#abs:6
**TXN --+> MMP2**	Regulation	info:pmid/11168376#abs:3
**TXN ---| TIMP1**	Regulation	info:pmid/11168376#abs:2
**TXN ---| TIMP1**	Regulation	info:pmid/14871484#body:109
**TXN ---| TIMP1**	Regulation	info:pmid/18639654#body:136
**TXN ---| TIMP1**	Regulation	info:doi/10.1016/j.yexcr.2003.07.005#body:72
**SPP1 --+> TIMP1**	Expression	info:pmid/17045020#abs:13
**SPP1 --+> TIMP1**	Expression	info:pmid/16128620#abs:8
**SPP1 --+> TIMP1**	Expression	info:pmid/21514415#abs:5
**SPP1 --+> TIMP1**	Expression	info:pmid/20595097#cont:241
**SPP1 --+> TIMP1**	Expression	info:pmid/21545755#cont:331
**SPP1 --+> TIMP1**	Expression	info:pmid/21037327#cont:2
**SPP1 --+> TIMP1**	Expression	info:pmid/21037326#body:90
**SPP1 --+> TIMP1**	MolTransport	info:pmid/20595097#abs:8
**ALB ---> HP**	Regulation	info:pmid/6984657#abs:4
**ALB ---> HP**	Regulation	info:pmid/20643827#abs:5
**ALB ---> HP**	Regulation	info:pmid/15504571#body:102
**HP ---| ALB**	Regulation	info:pmid/23564505#abs:7
**HP ---| ALB**	Regulation	info:pmid/14511687#body:130
**HP ---| ALB**	Regulation	info:pmid/19659435#cont:25
**HP ---| ALB**	Regulation	info:pmid/10400306#body:38
**HP ---| ALB**	Regulation	info:pmid/16778276#body:148
**DSTN ---> CFL1**	Regulation	info:pmid/19539025#body:77
**DSTN ---> CFL1**	Regulation	info:pmid/8674111

<table>
<tr><td>ALB --+> AK2</td><td>Regulation</td><td>info:pmid/12034470#body:110</td></tr>
<tr><td>FN1 --+> SPP1</td><td>Expression</td><td>info:pmid/16963119#body:122</td></tr>
<tr><td>FN1 --+> SPP1</td><td>Expression</td><td>info:pmid/16154538#body:20</td></tr>
<tr><td>FN1 --+> SPP1</td><td>Expression</td><td>info:pmid/10845864#body:166</td></tr>
<tr><td>FN1 --+> SPP1</td><td>Expression</td><td>info:pmid/11519777#body:276</td></tr>
<tr><td>FN1 --+> SPP1</td><td>Expression</td><td>info:doi/10.1016/j.archoralbio.2009.12.003#body:74</td></tr>
<tr><td>SPP1 --+> FN1</td><td>DirectRegulation</td><td>info:pmid/15770497#abs:2</td></tr>
<tr><td>SPP1 --+> FN1</td><td>DirectRegulation</td><td>info:pmid/20130530#abs:6</td></tr>
<tr><td>SPP1 --+> FN1</td><td>DirectRegulation</td><td>info:pmid/12473670#body:75</td></tr>
<tr><td>SPP1 --+> FN1</td><td>DirectRegulation</td><td>info:pmid/15548383#body:12</td></tr>
<tr><td>SPP1 --+> FN1</td><td>DirectRegulation</td><td>info:pmid/7785895#title:1</td></tr>
<tr><td>SPP1 --+> FN1</td><td>DirectRegulation</td><td>info:pmid/11564733#body:39</td></tr>
<tr><td>SPP1 --+> FN1</td><td>DirectRegulation</td><td>info:pmid/19573532#body:144</td></tr>
<tr><td>SPP1 --+> FN1</td><td>DirectRegulation</td><td>info:pmid/8941637#body:327</td></tr>
<tr><td>SPP1 --+> FN1</td><td>DirectRegulation</td><td>info:pmid/15247285#body:63</td></tr>
<tr><td>SPP1 --+> FN1</td><td>DirectRegulation</td><td>info:pmid/18490187#body:124</td></tr>
<tr><td>SPP1 --+> FN1</td><td>DirectRegulation</td><td>info:pmid/17110065#body:106</td></tr>
<tr><td>SPP1 --+> FN1</td><td>DirectRegulation</td><td>info:pmid/16125142#body:6</td></tr>
<tr><td>SPP1 --+> FN1</td><td>DirectRegulation</td><td>info:pmid/20138033#body:7</td></tr>
<tr><td>SPP1 --+> FN1</td><td>DirectRegulation</td><td>info:pmid/22836041#body:92</td></tr>
<tr><td>SPP1 --+> FN1</td><td>DirectRegulation</td><td>info:pmid/11696588#body:323</td></tr>
<tr><td>SPP1 --+> FN1</td><td>DirectRegulation</td><td>info:pmid/18314188#body:12</td></tr>
<tr><td>SPP1 --+> FN1</td><td>DirectRegulation</td><td>info:pmid/24361865#cont:314</td></tr>
<tr><td>SPP1 --+> FN1</td><td>DirectRegulation</td><td>info:pmid/21104439#cont:177</td></tr>
<tr><td>SPP1 --+> FN1</td><td>DirectRegulation</td><td>info:pmid/20127409#cont:44</td></tr>
<tr><td>SPP1 --+> FN1</td><td>DirectRegulation</td><td>info:pmid/16678698#body:80</td></tr>
<tr><td>SPP1 --+> FN1</td><td>DirectRegulation</td><td>info:pmid/11854297#body:322</td></tr>
<tr><td>SPP1 --+> FN1</td><td>DirectRegulation</td><td>info:pmid/15120833#body:71</td></tr>
<tr><td>SPP1 --+> FN1</td><td>DirectRegulation</td><td>info:pmid/18645046#body:130</td></tr>
<tr><td>SPP1 --+> FN1</td><td>DirectRegulation</td><td>info:pmid/11375279#body:73</td></tr>
<tr><td>SPP1 --+> FN1</td><td>DirectRegulation</td><td>info:pmid/15117918#body:42</td></tr>
<tr><td>SPP1 --+> FN1</td><td>DirectRegulation</td><td>info:pmid/15123578#body:56</td></tr>
<tr><td>SPP1 --+> FN1</td><td>DirectRegulation</td><td>info:doi/10.1016/j.pharmthera.2011.12.010#body:321</td></tr>
<tr><td>HP ---| CTSB</td><td>Regulation</td><td>info:pmid/7116206#abs:1</td></tr>
<tr><td>HP ---| CTSB</td><td>Regulation</td><td>info:pmid/7407678#abs:3</td></tr>
<tr><td>HP ---| CTSB</td><td>Regulation</td><td>info:pmid/10960776#body:139</td></tr>
<tr><td>HP ---| CTSB</td><td>Regulation</td><td>info:pmid/19074141#body:68</td></tr>
<tr><td>HP ---| CTSB</td><td>Regulation</td><td>info:pmid/7250307#title:1</td></tr>
<tr><td>HP ---| CTSB</td><td>Regulation</td><td>info:pmid/19306859#body:155</td></tr>
<tr><td>HP ---| CTSB</td><td>Regulation</td><td>info:pmid/17198677#body:97</td></tr>
<tr><td>ALB --+> FN1</td><td>Expression</td><td>info:pmid/17226761#abs:12</td></tr>
<tr><td>ALB --+> FN1</td><td>Expression</td><td>info:pmid/3682037#abs:4</td></tr>
<tr><td>ALB --+> FN1</td><td>Expression</td><td>info:pmid/19893244#abs:1</td></tr>
<tr><td>ALB --+> FN1</td><td>Expression</td><td>info:pmid/17048651#abs:9</td></tr>
<tr><td>ALB --+> FN1</td><td>Expression</td><td>info:pmid/16316526#abs:11</td></tr>
<tr><td>ALB --+> FN1</td><td>Expression</td><td>info:pmid/10361858#body:296</td></tr>
<tr><td>ALB --+> FN1</td><td>Expression</td><td>info:pmid/10916091#body:55</td></tr>
<tr><td>ALB --+> FN1</td><td>Expression</td><td>info:pmid/21270762#cont:230</td></tr>
<tr><td>ALB --+> FN1</td><td>Expression</td><td>info:pmid/20960212#cont:71</td></tr>
<tr><td>ALB ---> FN1</td><td>DirectRegulation</td><td>info:pmid/12623152#abs:12</td></tr>
</table>

**ALB ---> FN1**	DirectRegulation	info:pmid/11278018#abs:5
**ALB ---> FN1**	DirectRegulation	info:pmid/19743815#abs:3
**ALB ---> FN1**	DirectRegulation	info:pmid/17518603#abs:7
**ALB ---> FN1**	DirectRegulation	info:pmid/3391237#abs:2
**ALB ---> FN1**	DirectRegulation	info:pmid/15835174#abs:3
**ALB ---> FN1**	DirectRegulation	info:pmid/17607748#abs:8
**ALB ---> FN1**	DirectRegulation	info:pmid/18952882#abs:7
**ALB ---> FN1**	DirectRegulation	info:pmid/23156739#abs:7
**ALB ---> FN1**	DirectRegulation	info:pmid/19893244#abs:11
**ALB ---> FN1**	DirectRegulation	info:pmid/11426429#abs:13
**ALB ---> FN1**	DirectRegulation	info:pmid/20466081#body:27
**ALB ---> FN1**	DirectRegulation	info:pmid/21723964#body:12
**ALB ---> FN1**	DirectRegulation	info:pmid/17239539#body:99
**ALB ---> FN1**	DirectRegulation	info:pmid/8963717#body:72
**ALB ---> FN1**	DirectRegulation	info:pmid/17580303#body:308
**ALB ---> FN1**	DirectRegulation	info:pmid/18442700#body:42
**ALB ---> FN1**	DirectRegulation	info:pmid/19822362#body:186
**ALB ---> FN1**	DirectRegulation	info:pmid/18676139#body:114
**ALB ---> FN1**	DirectRegulation	info:pmid/15976454#body:66
**ALB ---> FN1**	DirectRegulation	info:pmid/16143139#body:83
**ALB ---> FN1**	DirectRegulation	info:pmid/22868193#body:184
**ALB ---> FN1**	DirectRegulation	info:pmid/23457620#cont:87
**ALB ---> FN1**	DirectRegulation	info:pmid/24092432#cont:305
**ALB ---> FN1**	DirectRegulation	info:pmid/20131932#cont:237
**ALB ---> FN1**	DirectRegulation	info:pmid/15102830#body:194
**ALB ---> FN1**	DirectRegulation	info:pmid/16567085#body:150
**ALB ---> FN1**	DirectRegulation	info:pmid/8977460#body:31
**ALB ---> FN1**	DirectRegulation	info:pmid/23523061#body:39
**ALB ---> FN1**	DirectRegulation	info:pmid/21238567#body:68
**ALB ---> FN1**	DirectRegulation	info:pmid/10521465#body:257
**ALB ---> FN1**	DirectRegulation	info:pmid/12778079#body:141
**ALB ---> FN1**	DirectRegulation	info:pmid/19124153#body:166
**ALB ---> FN1**	DirectRegulation	info:pmid/22211116#cont:104
**ALB ---> FN1**	DirectRegulation	info:pmid/11792823#body:164
**ALB ---> FN1**	DirectRegulation	info:pmid/20692384#body:158
**ALB ---> FN1**	DirectRegulation	info:pmid/16125545#body:418
**ALB ---> FN1**	DirectRegulation	info:pmid/14987565#body:25
**ALB ---> FN1**	DirectRegulation	info:doi/10.1016/j.bpj.2011.08.029#body:45
**FN1 ---| ALB**	Regulation	info:pmid/19815730#cont:60
**FN1 ---| ALB**	Regulation	info:pmid/18086444#body:88
**FN1 ---| ALB**	Regulation	info:pmid/15743489#body:147
**FN1 ---| ALB**	Regulation	info:pmid/23482756#cont:69
**FN1 ---| ALB**	Regulation	info:pmid/18261887#body:128
**FN1 ---| ALB**	Regulation	info:pmid/23707849#body:103
**FN1 ---| ALB**	Regulation	info:pmid/18164258#body:44
**FN1 ---| ALB**	Regulation	info:pmid/19767110#body:58
**FN1 ---| ALB**	Regulation	info:pmid/19744702#body:71
**FN1 ---| ALB**	Regulation	info:pmid/16023839#body:196
**FN1 ---| ALB**	Regulation	info:pmid/18784251#body:87
**FN1 ---| ALB**	Regulation	info:pmid/22378540#cont:215

**FN1 ---| ALB**	Regulation	info:doi/10.1016/j.biomaterials.2003.08.027#body:140
**AGT --+> LAMB1**	Regulation	info:pmid/15588708#body:97
**SPP1 --+> MMP2**	Expression	info:pmid/11564733#abs:4
**SPP1 --+> MMP2**	Expression	info:pmid/16077399#abs:8
**SPP1 --+> MMP2**	Expression	info:pmid/16474180#abs:12
**SPP1 --+> MMP2**	Expression	info:pmid/21174062#abs:7
**SPP1 --+> MMP2**	Expression	info:pmid/20207476#abs:7
**SPP1 --+> MMP2**	Expression	info:pmid/15548383#body:140
**SPP1 --+> MMP2**	Expression	info:pmid/14500723#body:148
**SPP1 --+> MMP2**	Expression	info:pmid/21785824#cont:33
**SPP1 --+> MMP2**	Expression	info:pmid/12114325#body:164
**SPP1 --+> MMP2**	Expression	info:pmid/21512310#cont:273
**SPP1 --+> MMP2**	Expression	info:pmid/21909361#cont:138
**SPP1 --+> MMP2**	Expression	info:pmid/19389394#body:93
**SPP1 --+> MMP2**	Expression	info:pmid/20689445#cont:172
**SPP1 --+> MMP2**	Expression	info:pmid/23652051#cont:148
**SPP1 --+> MMP2**	Expression	info:pmid/15579312#body:102
**SPP1 --+> MMP2**	Expression	info:pmid/24071501#body:109
**SPP1 --+> MMP2**	Expression	info:pmid/15501463#body:96
**SPP1 --+> MMP2**	Expression	info:pmid/22391222#body:404
**SPP1 --+> MMP2**	Expression	info:pmid/11964409#body:330
**SPP1 --+> MMP2**	Expression	info:pmid/22471890#cont:550
**SPP1 --+> MMP2**	Expression	info:pmid/22292748#cont:65
**SPP1 --+> MMP2**	Expression	info:pmid/12473670#body:455
**SPP1 --+> MMP2**	Expression	info:pmid/17187779#body:25
**SPP1 --+> MMP2**	Expression	info:pmid/21660447#cont:170
**SPP1 --+> MMP2**	Expression	info:pmid/23992637#body:109
**SPP1 --+> MMP2**	Expression	info:pmid/16631740#body:300
**SPP1 --+> MMP2**	Expression	info:pmid/20459645#body:108
**SPP1 --+> MMP2**	Expression	info:pmid/16728711#body:65
**SPP1 --+> MMP2**	Expression	info:pmid/22370646#cont:157
**SPP1 --+> MMP2**	Expression	info:doi/10.1016/j.freeradbiomed.2012.02.035#body:404
**ALB ---> AGT**	Regulation	info:pmid/2019263#abs:2
**ALB ---> AGT**	Regulation	info:pmid/623774#abs:5
**ALB ---> AGT**	Regulation	info:pmid/21558957#cont:80
**ALB ---> AGT**	Regulation	info:pmid/19141296#body:138
**ALB ---> AGT**	Regulation	info:pmid/12444210#body:292
**ALB ---> AGT**	Regulation	info:pmid/21659821#cont:42
**ALB ---> AGT**	Regulation	info:pmid/10405203#body:121
**AGT --+> ALB**	MolTransport	info:pmid/16293694#abs:4
**AGT --+> ALB**	MolTransport	info:pmid/12887133#abs:9
**AGT --+> ALB**	MolTransport	info:pmid/7150395#abs:5
**AGT --+> ALB**	MolTransport	info:pmid/19082426#abs:6
**AGT --+> ALB**	MolTransport	info:pmid/14757134#abs:3
**AGT --+> ALB**	MolTransport	info:pmid/22610172#abs:7
**AGT --+> ALB**	MolTransport	info:pmid/21415394#cont:179
**AGT --+> ALB**	MolTransport	info:pmid/16256138#body:84
**AGT --+> ALB**	MolTransport	info:pmid/20359530#body:78
**HP ---> CP**	Regulation	info:doi/10.1016/j.cca.2011.02.003#body:110
**CTSB ---> MMP2**	Expression	info:pmid/21854877#body:112

**CTSB ---> MMP2**	Expression	info:pmid/23811845#cont:46
**SOD2 --+> MMP2**	Regulation	info:pmid/11929863#abs:7
**SOD2 --+> MMP2**	Regulation	info:pmid/20479714#cont:174
**SOD2 --+> MMP2**	Regulation	info:pmid/21238464#body:133
**SOD2 --+> MMP2**	Regulation	info:pmid/22695858#cont:37
**SOD2 --+> MMP2**	Regulation	info:pmid/16569638#body:383
**SOD2 --+> MMP2**	Regulation	info:pmid/23583431#body:19
**SOD2 --+> MMP2**	Regulation	info:pmid/21434856#cont:738
**SOD2 --+> MMP2**	Regulation	info:pmid/16430879#body:599
**SOD2 --+> MMP2**	Regulation	info:pmid/15304253#body:118
**SOD2 --+> MMP2**	Regulation	info:pmid/17597617#body:120
**SOD2 --+> MMP2**	Regulation	info:pmid/21345984#cont:162
**CFL1 --+> MMP2**	Expression	info:pmid/16337627#abs:9
**CFL1 --+> MMP2**	Expression	info:pmid/18499298#body:261
**TXN ---| LGALS3**	Regulation	info:pmid/14644168#abs:5
**TXN ---| LGALS3**	Regulation	info:doi/10.1016/j.yexcr.2003.07.005#body:89
**CTSB ---> TIMP1**	Expression	info:pmid/16469948#body:212
**CTSB ---> TIMP1**	Expression	info:pmid/22673002#body:225
**CTSB ---> TIMP1**	Expression	info:pmid/10437790#body:7
**CTSB ---> TIMP1**	Expression	info:pmid/21394106#cont:387
**TIMP1 ---| CTSB**	Regulation	info:pmid/20957082#cont:71
**LCN2 ---> FN1**	Expression	info:pmid/19237579#abs:3
**LCN2 ---> FN1**	Expression	info:pmid/24194573#cont:351
**LCN2 ---> FN1**	Expression	info:pmid/17949711#body:288
**LCN2 ---> FN1**	Expression	info:pmid/20403349#body:214
**LCN2 ---> FN1**	Expression	info:pmid/22075378#body:50
**AIMP1 ---| FN1**	Regulation	info:pmid/20212356#abs:8
**AIMP1 ---| FN1**	Regulation	info:pmid/16248999#abs:5
**AIMP1 ---| FN1**	Regulation	info:pmid/20616358#cont:196
**AIMP1 ---| FN1**	Regulation	info:pmid/19002109#body:234
**AIMP1 ---| FN1**	Regulation	info:pmid/22723862#cont:109
**AIMP1 ---| FN1**	Regulation	info:pmid/19628080#body:89
**SOD2 ---| ALB**	Regulation	info:pmid/14739156#body:168
**ALB ---> SOD2**	Regulation	info:pmid/21035442#body:65
**TXN --+> SOD2**	Expression	info:pmid/9409558#abs:5
**TXN --+> SOD2**	Expression	info:pmid/15337734#body:93
**TXN --+> SOD2**	Expression	info:pmid/21120747#cont:369
**TXN --+> SOD2**	Expression	info:pmid/21731078#cont:182
**TXN --+> SOD2**	Expression	info:pmid/11751890#body:151
**TXN --+> SOD2**	Expression	info:pmid/12788387#body:145
**TXN --+> SOD2**	Expression	info:pmid/24253037#cont:315
**TXN --+> SOD2**	Expression	info:pmid/18206121#body:52
**TXN --+> SOD2**	Expression	info:pmid/15894008#body:130
**TXN --+> SOD2**	Expression	info:pmid/11035260#body:79
**TXN --+> SOD2**	Expression	info:pmid/23025925#body:201
**TXN --+> SOD2**	Expression	info:pmid/16815478#body:8
**TXN --+> SOD2**	Expression	info:pmid/24023246#cont:463
**TXN --+> SOD2**	Expression	info:pmid/12122214#body:51
**TXN --+> SOD2**	Expression	info:pmid/15277664#body:185
**TXN --+> SOD2**	Expression	info:pmid/20188820#body:198

**TXN --+> SOD2**	Expression	info:pmid/17210450#body:199
**TXN --+> SOD2**	Expression	info:pmid/16099847#body:238
**ALB ---| GNB2L1**	Regulation	info:pmid/22240482#body:33
**SPARC --+> SPP1**	Expression	info:pmid/16412713#body:110
**SPP1 ---> ALB**	DirectRegulation	info:pmid/21071960#abs:2
**SPP1 ---> ALB**	DirectRegulation	info:pmid/10751402#abs:5
**SPP1 ---> ALB**	DirectRegulation	info:pmid/8910476#body:119
**SPP1 ---> ALB**	DirectRegulation	info:pmid/22370646#cont:76
**SPP1 ---> ALB**	DirectRegulation	info:pmid/14987565#body:25
**CFL1 ---> GSN**	Expression	info:pmid/9079652#abs:8
**CFL1 ---> GSN**	Expression	info:pmid/18171679#body:106
**CFL1 ---> GSN**	Expression	info:pmid/18171680#body:106
**CFL1 ---> GSN**	Expression	info:pmid/15128850#body:413
**GSN ---> CFL1**	Expression	info:pmid/18171679#body:106
**GSN ---> CFL1**	Expression	info:pmid/18171680#body:106
**GSN ---> CFL1**	Expression	info:pmid/15128850#body:413
**ALB ---| TXN**	Regulation	info:pmid/21126209#abs:7
**ALB ---| TXN**	Regulation	info:pmid/24361613#body:149
**ALB ---| TXN**	Regulation	info:pmid/17395017#body:232
**TXN ---> ALB**	Regulation	info:pmid/22989946#body:189
**TXN ---> ALB**	Regulation	info:pmid/21126209#cont:173
**TXN ---> ALB**	Regulation	info:pmid/18206876#body:16
**TXN ---> ALB**	Regulation	info:doi/10.1016/j.foodchem.2011.09.022#body:107
**AGT --+> TIMP1**	Expression	info:pmid/12388255#abs:5
**AGT --+> TIMP1**	Expression	info:pmid/23796502#abs:8
**AGT --+> TIMP1**	Expression	info:pmid/12846741#abs:13
**AGT --+> TIMP1**	Expression	info:pmid/18272044#abs:15
**AGT --+> TIMP1**	Expression	info:pmid/18252761#abs:10
**AGT --+> TIMP1**	Expression	info:pmid/18756259#abs:9
**AGT --+> TIMP1**	Expression	info:pmid/18296491#abs:5
**AGT --+> TIMP1**	Expression	info:pmid/14551224#abs:4
**AGT --+> TIMP1**	Expression	info:pmid/12677092#abs:8
**AGT --+> TIMP1**	Expression	info:pmid/21646597#abs:7
**AGT --+> TIMP1**	Expression	info:pmid/12480812#body:181
**AGT --+> TIMP1**	Expression	info:pmid/17502491#body:175
**AGT --+> TIMP1**	Expression	info:pmid/8664344#title:1
**AGT --+> TIMP1**	Expression	info:pmid/22493071#cont:260
**AGT --+> TIMP1**	Expression	info:pmid/21152444#body:251
**AGT --+> TIMP1**	Expression	info:pmid/12522128#body:168
**AGT --+> TIMP1**	Expression	info:pmid/17884089#body:117
**AGT --+> TIMP1**	Expression	info:pmid/21450123#cont:406
**AGT --+> TIMP1**	Expression	info:pmid/24257335#cont:31
**AGT --+> TIMP1**	Expression	info:pmid/12819040#body:16
**AGT --+> TIMP1**	Expression	info:pmid/19233360#body:130
**AGT --+> TIMP1**	Expression	info:pmid/15331071#body:82
**AGT --+> TIMP1**	Expression	info:pmid/19460403#body:288
**AGT --+> TIMP1**	Expression	info:pmid/19027749#body:97
**AGT --+> TIMP1**	Expression	info:pmid/16834928#title:1
**AGT --+> TIMP1**	Expression	info:pmid/20881940#cont:36
**AGT --+> TIMP1**	Expression	info:pmid/17950364#body:154

**AGT --+> TIMP1**	Expression	info:pmid/18395779#body:96
**AGT --+> TIMP1**	Expression	info:pmid/21228800#cont:76
**AGT --+> TIMP1**	Expression	info:pmid/14569113#body:87
**AGT --+> TIMP1**	Expression	info:pmid/17395057#body:80
**AGT --+> TIMP1**	Expression	info:pmid/21395550#cont:436
**AGT --+> TIMP1**	Expression	info:pmid/21718678#body:146
**AGT --+> TIMP1**	Expression	info:pmid/21228799#cont:100
**AGT --+> TIMP1**	Expression	info:pmid/16139830#body:400
**AGT --+> TIMP1**	Expression	info:pmid/18619957#body:91
**AGT --+> TIMP1**	Expression	info:pmid/18579222#body:66
**AGT --+> TIMP1**	Expression	info:pmid/12912805#body:117
**AGT --+> TIMP1**	Expression	info:pmid/22846599#body:130
**AGT --+> TIMP1**	Expression	info:pmid/10856263#body:157
**AGT --+> TIMP1**	Expression	info:pmid/21298056#body:232
**AGT --+> TIMP1**	Expression	info:pmid/22073128#cont:160
**AGT --+> TIMP1**	Expression	info:pmid/15860759#body:195
**AGT --+> TIMP1**	Expression	info:pmid/11244005#body:40
**AGT --+> TIMP1**	Expression	info:doi/10.1016/j.yjmcc.2004.12.015#body:126
**AGT --+> TIMP1**	Expression	info:doi/10.1016/S0025-7125(03)00125-1#body:94
**FN1 ---| SERPINC1**	Regulation	info:pmid/3858983#abs:2
**FN1 ---| SERPINC1**	Regulation	info:pmid/9585886#body:58
**FN1 ---| SERPINC1**	Regulation	info:pmid/10475283#body:189
**FN1 ---| SERPINC1**	Regulation	info:pmid/21463893#body:324
**FN1 ---| SERPINC1**	Regulation	info:pmid/20692134#body:119
**FN1 ---| SERPINC1**	Regulation	info:pmid/10605952#body:4
**FN1 ---| SERPINC1**	Regulation	info:pmid/21805447#cont:152
**ALB ---> SERPINC1**	Regulation	info:pmid/531528#abs:7
**ALB ---> SERPINC1**	Regulation	info:pmid/19442540#body:35
**ALB ---> SERPINC1**	Regulation	info:doi/10.1016/S0741-5214(97)70271-4#body:3
**PPIA --+> MMP2**	Expression	info:pmid/19789967#abs:4
**PPIA --+> MMP2**	Expression	info:pmid/19430489#abs:7
**PPIA --+> MMP2**	Expression	info:pmid/19968957#body:79
**PPIA --+> MMP2**	Expression	info:pmid/20589525#cont:166
**PPIA --+> MMP2**	Expression	info:pmid/23031673#cont:230
**PPIA --+> MMP2**	Expression	info:pmid/20043978#body:77
**PPIA --+> MMP2**	Expression	info:pmid/20598751#body:219
**PPIA --+> MMP2**	Expression	info:pmid/21441138#cont:46
**PPIA --+> MMP2**	MolTransport	info:pmid/19789967#abs:6
**PPIA --+> MMP2**	MolTransport	info:pmid/23167819#cont:96
**PPIA ---| PRDX4**	Regulation	info:pmid/16321424#body:7
**SPP1 --+> LCN2**	Regulation	info:pmid/22366155#abs:6
**SPP1 --+> LCN2**	Regulation	info:doi/10.1016/j.taap.2012.02.006#body:188
**TXN ---| PRDX4**	Regulation	info:pmid/20682242#body:541
**TXN ---| PRDX4**	Regulation	info:pmid/16321424#body:7
**HP ---> TF**	DirectRegulation	info:pmid/2150414#abs:3
**HP ---> TF**	DirectRegulation	info:pmid/16209357#abs:2
**HP ---> TF**	DirectRegulation	info:pmid/17008602#body:178
**HP ---> TF**	DirectRegulation	info:pmid/23927895#body:2
**HP ---> TF**	DirectRegulation	info:pmid/16778276#body:148
**HP ---> TF**	DirectRegulation	info:pmid/17367871#body:55

**PEBP1 --+> GNB2L1**	Regulation	info:pmid/19545586#body:144
**CTSB --+> PRSS1**	Regulation	info:pmid/16534247#abs:11
**CTSB --+> PRSS1**	Regulation	info:pmid/16492714#abs:3
**CTSB --+> PRSS1**	Regulation	info:pmid/11932257#abs:5
**CTSB --+> PRSS1**	Regulation	info:pmid/14507909#body:271
**CTSB --+> PRSS1**	Regulation	info:pmid/16632095#body:196
**CTSB --+> PRSS1**	Regulation	info:pmid/15582989#body:218
**CTSB --+> PRSS1**	Regulation	info:pmid/19801634#body:194
**IGFBP7 --+> PEBP1**	Regulation	info:pmid/18267069#body:116
**AGT --+> ANGPT2**	Expression	info:pmid/14638905#abs:7
**AGT --+> ANGPT2**	Expression	info:pmid/22613986#abs:9
**AGT --+> ANGPT2**	Expression	info:pmid/11289054#abs:7
**AGT --+> ANGPT2**	Expression	info:pmid/20056745#abs:4
**AGT --+> ANGPT2**	Expression	info:pmid/20556927#cont:38
**AGT --+> ANGPT2**	Expression	info:pmid/21829546#cont:39
**AGT --+> ANGPT2**	Expression	info:pmid/11139469#body:80
**AGT --+> ANGPT2**	Expression	info:pmid/23838360#body:25
**AGT --+> ANGPT2**	Expression	info:pmid/12937129#body:103
**AGT --+> ANGPT2**	Expression	info:pmid/12745003#body:165
**AGT --+> ANGPT2**	Expression	info:pmid/15583069#body:54
**AGT --+> ANGPT2**	Expression	info:pmid/16288810#body:135
**AGT --+> ANGPT2**	Expression	info:pmid/12110003#body:406
**AGT --+> ANGPT2**	Expression	info:pmid/17065527#body:224
**AGT --+> ANGPT2**	Expression	info:doi/10.1016/j.bbrc.2003.08.086#body:71
**LAMC1 ---> LAMA2**	DirectRegulation	info:pmid/14638863#abs:4
**LAMC1 ---> LAMA2**	DirectRegulation	info:pmid/12506770#body:89
**LAMC1 ---> LAMA2**	DirectRegulation	info:pmid/23897819#cont:229
**LAMC1 ---> LAMA2**	DirectRegulation	info:pmid/20067997#body:310
**SOD2 ---| SOD3**	Expression	info:pmid/16195479#body:185
**SOD2 ---| SOD3**	Expression	info:pmid/20393169#cont:187
**SOD3 ---> SOD2**	Expression	info:pmid/20932897#body:110
**SOD3 ---> SOD2**	Expression	info:pmid/20096764#body:17
**SOD3 ---> SOD2**	Expression	info:pmid/12816884#body:141
**SOD3 ---> SOD2**	Expression	info:pmid/12471025#body:149
**SOD3 ---> SOD2**	Expression	info:pmid/22247605#cont:234
**SOD2 ---| SOD3**	DirectRegulation	info:pmid/20932897#abs:2
**SOD2 ---| SOD3**	DirectRegulation	info:pmid/23341968#cont:505
**SOD2 ---| SOD3**	DirectRegulation	info:pmid/18594523#body:93
**SOD2 ---| SOD3**	DirectRegulation	info:pmid/19819949#body:275
**SOD2 ---| SOD3**	DirectRegulation	info:pmid/19439219#body:29
**SOD2 ---| SOD3**	DirectRegulation	info:pmid/20079425#body:63
**SOD2 ---| SOD3**	DirectRegulation	info:pmid/21530649#body:152
**SOD2 ---| SOD3**	DirectRegulation	info:pmid/23299361#cont:107
**SOD2 ---| SOD3**	DirectRegulation	info:pmid/16716903#body:4
**SOD2 ---| SOD3**	DirectRegulation	info:pmid/18406553#body:177
**AGT --+> SOD3**	Expression	info:pmid/22753205#abs:2
**AGT --+> SOD3**	Expression	info:pmid/10400907#abs:9
**AGT --+> SOD3**	Expression	info:pmid/11557552#abs:4
**AGT --+> SOD3**	Expression	info:pmid/15337734#body:186
**AGT --+> SOD3**	Expression	info:pmid/17015770#body:171

**AGT --+> SOD3**	Expression	info:pmid/16857181#body:73
**AGT --+> SOD3**	Expression	info:pmid/20919937#cont:252
**AGT --+> SOD3**	Expression	info:pmid/19059306#body:131
**AGT --+> SOD3**	Expression	info:pmid/18695146#body:137
**AGT --+> SOD3**	Expression	info:pmid/18768397#body:110
**AGT --+> SOD3**	Expression	info:pmid/23628292#body:134
**AGT --+> SOD3**	Expression	info:pmid/23934707#cont:159
**AGT --+> SOD3**	Expression	info:pmid/12816884#body:59
**AGT --+> SOD3**	Expression	info:pmid/11557673#body:33
**AGT --+> SOD3**	Expression	info:doi/10.1016/j.jash.2006.11.006#body:50
**AGT --+> SOD3**	Expression	info:doi/10.1016/j.semnephrol.2003.11.008#body:64
**XDH --+> MMP2**	Regulation	info:pmid/15841469#abs:9
**XDH --+> MMP2**	Regulation	info:pmid/15304253#body:145
**XDH --+> MMP2**	Regulation	info:pmid/8958220#body:191
**AGT --+> PPIA**	MolTransport	info:pmid/24293519#abs:8
**AGT --+> PPIA**	MolTransport	info:pmid/23846495#abs:8
**AGT --+> PPIA**	MolTransport	info:pmid/21934628#cont:111
**AGT --+> PPIA**	MolTransport	info:pmid/21330604#cont:279
**AGT --+> PPIA**	MolTransport	info:pmid/24176846#cont:95
**AGT --+> PPIA**	MolTransport	info:pmid/23669100#body:49
**CTSB ---> AGT**	Expression	info:pmid/3911093#abs:6
**CTSB ---> AGT**	Expression	info:pmid/16697369#body:120
**AGT --+> CTSB**	Regulation	info:pmid/21964156#cont:201
**AGT --+> CTSB**	Regulation	info:pmid/23226500#cont:91
**FN1 ---> TIMP1**	Expression	info:pmid/22516051#abs:3
**FN1 ---> TIMP1**	Expression	info:pmid/20821288#cont:212
**FN1 ---> TIMP1**	Expression	info:pmid/11420245#body:229
**FN1 ---> TIMP1**	Expression	info:pmid/17395008#body:134
**FN1 ---> TIMP1**	Expression	info:pmid/15615902#body:176
**FN1 ---> TIMP1**	Expression	info:pmid/12700193#body:235
**FN1 ---> TIMP1**	Expression	info:doi/10.3727/096504011X13079697132925#cont:390
**FN1 ---> TIMP1**	Expression	info:doi/10.2353/ajpath.2007.060456#body:155
**LAMB1 --+> MMP2**	Expression	info:pmid/15036260#body:36
**LAMB1 --+> MMP2**	Expression	info:pmid/15781322#body:94
**XDH ---| DCXR**	Regulation	info:pmid/11731027#body:13
**FN1 --+> SPARC**	Expression	info:pmid/12468382#abs:10
**FN1 --+> SPARC**	Expression	info:pmid/12655283#abs:7
**FN1 --+> SPARC**	Expression	info:pmid/22733805#cont:168
**FN1 --+> SPARC**	Expression	info:pmid/24223185#cont:235
**FN1 --+> SPARC**	Expression	info:pmid/18820161#body:245
**FN1 --+> SPARC**	Expression	info:pmid/16115889#body:266
**SPARC --+> FN1**	Expression	info:pmid/1379603#abs:4
**SPARC --+> FN1**	Expression	info:pmid/22136411#abs:5
**SPARC --+> FN1**	Expression	info:pmid/17056239#body:83
**SPARC --+> FN1**	Expression	info:pmid/19958839#body:161
**SPARC --+> FN1**	Expression	info:pmid/16685412#cont:169
**SPARC --+> FN1**	Expression	info:pmid/22884391#body:131
**SPARC --+> FN1**	Expression	info:pmid/15976454#body:203
**SPARC --+> FN1**	DirectRegulation	info:pmid/21609765#abs:1
**SPARC --+> FN1**	DirectRegulation	info:pmid/21311029#abs:6

**SPARC --+> FN1**	DirectRegulation	info:pmid/16115889#abs:6
**SPARC --+> FN1**	DirectRegulation	info:pmid/24223185#cont:212
**SPARC --+> FN1**	DirectRegulation	info:pmid/18216330#body:91
**SPARC --+> FN1**	DirectRegulation	info:pmid/21237573#body:89
**SPARC --+> FN1**	DirectRegulation	info:pmid/18990699#body:372
**SPARC --+> FN1**	DirectRegulation	info:pmid/23139787#cont:213
**SPARC --+> FN1**	DirectRegulation	info:pmid/22718352#cont:243
**SPARC --+> FN1**	DirectRegulation	info:pmid/19103879#body:187
**SPARC --+> FN1**	DirectRegulation	info:pmid/19958839#body:122
**SPARC --+> FN1**	DirectRegulation	info:pmid/15976454#body:241
**SPARC --+> FN1**	DirectRegulation	info:pmid/20811805#cont:347
**SPARC --+> FN1**	DirectRegulation	info:pmid/17490972#body:193
**SPARC --+> FN1**	DirectRegulation	info:pmid/14987565#body:85
**SPARC --+> FN1**	DirectRegulation	info:pmid/22070921#body:81
**ALB --+> XDH**	DirectRegulation	info:pmid/23021852#body:37
**ALB --+> XDH**	DirectRegulation	info:doi/10.1016/j.foodchem.2010.10.029#body:19
**ALB --+> XDH**	DirectRegulation	info:doi/10.1016/j.foodchem.2009.06.016#body:82
**ALB --+> XDH**	DirectRegulation	info:doi/10.1016/j.molcatb.2010.06.002#body:16
**XDH --+> ALB**	MolTransport	info:pmid/18423414#body:527
**XDH --+> ALB**	MolTransport	info:pmid/22154653#body:517
**CTSB ---| ALB**	Expression	info:pmid/7306078#abs:10
**CTSB ---| ALB**	Expression	info:pmid/17714804#body:6
**ALB ---> CTSB**	Regulation	info:pmid/12217416#body:43
**ALB ---> CTSB**	Regulation	info:pmid/9199883#body:139
**LAMB1 ---| LAMA2**	Regulation	info:pmid/23897819#cont:229
**ALB --+> LCN2**	MolTransport	info:pmid/23429046#abs:9
**HP ---| MMP2**	Regulation	info:pmid/17320799#body:110
**HP ---| MMP2**	Regulation	info:pmid/21296538#body:110
**TXN ---| PPIA**	Regulation	info:pmid/18331844#body:126
**TF ---> C3**	Expression	info:pmid/12223215#body:112
**TF ---> C3**	Expression	info:pmid/11136173#title:1
**TF ---> C3**	Expression	info:pmid/11967015#body:328
**C3 ---| TF**	Regulation	info:pmid/16778276#body:148
**FN1 --+> CYR61**	Regulation	info:pmid/19440550#body:421
**CYR61 --+> FN1**	Regulation	info:pmid/17698398#body:138
**ALB ---> MMP2**	Expression	info:pmid/17368046#body:85
**ALB ---> MMP2**	Expression	info:pmid/19933182#cont:149
**FN1 ---> CFL1**	Regulation	info:pmid/16125057#body:110
**FN1 ---> CFL1**	Regulation	info:pmid/11294912#body:270
**FN1 ---> CFL1**	Regulation	info:pmid/15866889#body:245
**FN1 ---> CFL1**	Regulation	info:pmid/19265031#body:136
**AGRN --+> LAMA2**	Regulation	info:pmid/12958164#abs:7
**AGRN --+> LAMA2**	Regulation	info:pmid/12921796#body:209
**LAMC1 ---| FN1**	Regulation	info:pmid/24576673#body:99
**SPP1 --+> GSN**	Regulation	info:pmid/8744948#abs:5
**SPP1 --+> GSN**	Regulation	info:pmid/11577104#body:445
**SPP1 --+> GSN**	Regulation	info:pmid/9565618#body:64
**SPP1 --+> GSN**	Regulation	info:pmid/9774337#body:35
**SPP1 --+> GSN**	Regulation	info:pmid/10047530#body:54
**SPP1 --+> GSN**	Regulation	info:pmid/16006560#body:228

**PRSS1 --+> TXN**	Regulation	info:pmid/14680968#body:123
**MMP2 --+> COL1A2**	Expression	info:doi/10.1016/j.jaci.2010.12.509#body:9
**COL1A2 --+> MMP2**	Regulation	info:doi/10.1016/j.matbio.2008.09.298#body:5
**AGT ---> TF**	DirectRegulation	info:pmid/16478976#abs:3
**AGT ---> TF**	DirectRegulation	info:pmid/20643827#abs:5
**AGT ---> TF**	DirectRegulation	info:pmid/20974581#abs:3
**AGT ---> TF**	DirectRegulation	info:pmid/11160859#body:133
**AGT ---> TF**	DirectRegulation	info:pmid/24184051#body:122
**TF ---> FN1**	Expression	info:pmid/16043859#body:293
**FN1 ---| TF**	Regulation	info:pmid/23707849#body:103
**ALB ---> LGALS3**	Expression	info:pmid/19493442#abs:8
**ALB ---> LGALS3**	Expression	info:pmid/23411399#body:89
**LGALS3 ---| ALB**	Regulation	info:pmid/23953881#body:59
**TIMP1 ---> CYR61**	Regulation	info:pmid/22079246#body:235
**CYR61 ---> TIMP1**	Regulation	info:pmid/22079246#body:235
**GSTP1 ---> LGALS3BP**	Regulation	info:doi/10.1016/j.jprot.2011.05.012#body:143
**PEBP1 ---> SPP1**	Expression	info:pmid/21873975#cont:322
**PEBP1 ---> SPP1**	Expression	info:pmid/22809510#body:76
**SPP1 ---| XDH**	Regulation	info:pmid/23639879#body:162
**AGT ---> GSTP1**	Regulation	info:pmid/24321768#body:125
**AGT --+> SPARC**	Expression	info:pmid/17717147#body:80
**AGT --+> SPARC**	Expression	info:pmid/23517551#cont:229
**SPARC ---> AGT**	Regulation	info:pmid/17717147#abs:3
**SOD2 ---> FN1**	Expression	info:pmid/21802526#body:223
**SOD2 ---> FN1**	Expression	info:pmid/21709278#cont:159
**SOD2 ---> FN1**	Expression	info:pmid/18083119#body:64
**AGT --+> THBS4**	Expression	info:pmid/18541142#abs:4
**AGT --+> THBS4**	Expression	info:pmid/17502491#body:162
**FN1 ---> GSN**	Expression	info:pmid/6092370#abs:5
**FN1 ---> GSN**	Expression	info:pmid/15922735#body:168
**FN1 ---> GSN**	DirectRegulation	info:pmid/6092370#abs:1
**FN1 ---> GSN**	DirectRegulation	info:pmid/19840195#abs:5
**FN1 ---> GSN**	DirectRegulation	info:pmid/10660524#abs:5
**FN1 ---> GSN**	DirectRegulation	info:pmid/21171974#cont:217
**FN1 ---> GSN**	DirectRegulation	info:pmid/15033991#body:204
**TCF3 ---> COL1A2**	Expression	info:pmid/19362560#body:163
**FN1 ---> CTSB**	Expression	info:pmid/23211306#title:1
**FN1 ---> CTSB**	Expression	info:pmid/15896466#body:105
**CTSB ---> FN1**	Expression	info:pmid/14764809#body:284
**CTSB ---> FN1**	Expression	info:pmid/12581740#body:5
**CYR61 --+> MMP2**	Expression	info:pmid/19632997#abs:7
**CYR61 --+> MMP2**	Expression	info:pmid/18187544#body:330
**MMP2 ---> CYR61**	Regulation	info:pmid/18187544#body:344
**CYR61 ---> MMP2**	MolTransport	info:pmid/22551568#body:93
**CYR61 ---> MMP2**	MolTransport	info:doi/10.1016/j.placenta.2012.04.005#body:93
**ALB ---> LAMB1**	Expression	info:pmid/21440603#body:122
**ALB ---> LAMB1**	Expression	info:pmid/12707408#body:99
**AGT ---| ENPP3**	Expression	info:pmid/12757929#body:96
**FN1 --+> LAMB1**	Expression	info:pmid/2015965#abs:4

<table>
<tr><td>LAMB1 ---> FN1</td><td>Expression</td><td>info:pmid/10925241#body:199</td></tr>
<tr><td>AIP ---> FN1</td><td>Expression</td><td>info:pmid/23333462#body:191</td></tr>
<tr><td>AGT ---> SOD2</td><td>Expression</td><td>info:pmid/20596034#abs:5</td></tr>
<tr><td>AGT ---> SOD2</td><td>Expression</td><td>info:pmid/22443458#cont:197</td></tr>
<tr><td>AGT ---> SOD2</td><td>Expression</td><td>info:pmid/22671943#cont:114</td></tr>
<tr><td>AGT ---> SOD2</td><td>Expression</td><td>info:pmid/23472618#cont:133</td></tr>
<tr><td>AGT ---> SOD2</td><td>Expression</td><td>info:pmid/12040026#body:82</td></tr>
<tr><td>AGT ---> SOD2</td><td>Expression</td><td>info:pmid/23348709#body:129</td></tr>
<tr><td>AGT ---> SOD2</td><td>Expression</td><td>info:pmid/14569087#body:237</td></tr>
<tr><td>AGT ---> SOD2</td><td>Expression</td><td>info:pmid/15166009#body:131</td></tr>
<tr><td>TCF3 --+> SPARC</td><td>Expression</td><td>info:pmid/22406545#body:65</td></tr>
<tr><td>TCF3 --+> SPARC</td><td>Expression</td><td>info:doi/10.1016/j.semcancer.2012.02.013#body:65</td></tr>
<tr><td>XDH --+> SOD2</td><td>Expression</td><td>info:pmid/21091077#cont:174</td></tr>
<tr><td>XDH --+> SOD2</td><td>Expression</td><td>info:pmid/9741582#body:220</td></tr>
<tr><td>SOD2 ---| XDH</td><td>Regulation</td><td>info:pmid/10318800#body:126</td></tr>
<tr><td>ANGPT2 --+> TIMP1</td><td>Expression</td><td>info:doi/10.1016/j.jaad.2003.10.122#body:15</td></tr>
<tr><td>PEBP1 ---> MMP2</td><td>Expression</td><td>info:pmid/20855151#abs:4</td></tr>
<tr><td>DCXR ---| MMP2</td><td>MolTransport</td><td>info:pmid/21824723#body:128</td></tr>
<tr><td>CFL1 --+> ALB</td><td>MolTransport</td><td>info:pmid/12904289#abs:8</td></tr>
<tr><td>CFL1 --+> ALB</td><td>MolTransport</td><td>info:pmid/23352899#body:131</td></tr>
<tr><td>CFL1 --+> ALB</td><td>MolTransport</td><td>info:pmid/16601121#body:79</td></tr>
<tr><td>CFL1 --+> ALB</td><td>MolTransport</td><td>info:pmid/21971085#cont:711</td></tr>
<tr><td>TCF3 ---> TF</td><td>PromoterBinding</td><td>info:pmid/18768914#body:305</td></tr>
<tr><td>AGT --+> CFL1</td><td>Regulation</td><td>info:pmid/21188136#abs:6</td></tr>
<tr><td>AGT --+> CFL1</td><td>Regulation</td><td>info:pmid/19339277#body:78</td></tr>
<tr><td>S100A10 ---| TF</td><td>Regulation</td><td>info:pmid/13679511#body:272</td></tr>
<tr><td>GSN ---> ALB</td><td>Regulation</td><td>info:pmid/10799543#body:172</td></tr>
<tr><td>GSN ---> ALB</td><td>Regulation</td><td>info:pmid/12654637#body:124</td></tr>
<tr><td>TCF3 --+> PTF1A</td><td>Regulation</td><td>info:pmid/19887377#body:242</td></tr>
<tr><td>AGT --+> COL1A2</td><td>Expression</td><td>info:pmid/23646205#cont:179</td></tr>
<tr><td>AGT --+> COL1A2</td><td>Expression</td><td>info:pmid/19204184#body:186</td></tr>
<tr><td>AGT --+> COL1A2</td><td>Expression</td><td>info:pmid/14871417#body:243</td></tr>
<tr><td>SOD3 ---| ALB</td><td>Regulation</td><td>info:pmid/14739156#body:168</td></tr>
<tr><td>ANGPT2 ---> FN1</td><td>Regulation</td><td>info:pmid/22585576#cont:301</td></tr>
<tr><td>ANGPT2 ---> FN1</td><td>Regulation</td><td>info:pmid/19553662#body:280</td></tr>
<tr><td>SPARC ---> COL1A2</td><td>Expression</td><td>info:pmid/21978691#abs:7</td></tr>
<tr><td>SPARC ---> COL1A2</td><td>Expression</td><td>info:pmid/18301770#body:184</td></tr>
<tr><td>COL1A2 ---> SPARC</td><td>Expression</td><td>info:pmid/18820161#body:245</td></tr>
<tr><td>GPI ---> FN1</td><td>DirectRegulation</td><td>info:pmid/16141236#abs:5</td></tr>
<tr><td>GPI ---> FN1</td><td>DirectRegulation</td><td>info:pmid/12888574#abs:4</td></tr>
<tr><td>AGT --+> S100A10</td><td>Regulation</td><td>info:pmid/12522128#body:171</td></tr>
<tr><td>TXN --+> GSTP1</td><td>Expression</td><td>info:pmid/17823364#abs:12</td></tr>
<tr><td>TXN ---> FKBP1A</td><td>Regulation</td><td>info:pmid/12923164#body:204</td></tr>
<tr><td>GNB2L1--->COL1A2</td><td>Expression</td><td>info:pmid/16849317#abs:12</td></tr>
<tr><td>GNB2L1 ---| TF</td><td>Regulation</td><td>info:pmid/11607837#body:132</td></tr>
<tr><td>AGT ---> TXN</td><td>Expression</td><td>info:pmid/17563540#abs:8</td></tr>
<tr><td>AGT ---> TXN</td><td>Expression</td><td>info:pmid/22828084#cont:129</td></tr>
<tr><td>AGT ---> TXN</td><td>Expression</td><td>info:pmid/22900666#cont:357</td></tr>
<tr><td>AGT ---> TXN</td><td>Expression</td><td>info:pmid/19506101#body:181</td></tr>
<tr><td>TXN ---| AGT</td><td>Regulation</td><td>info:pmid/15123525#body:126</td></tr>
</table>

**ALB --+> GALNS**	Regulation	info:pmid/17611269#body:195
**LAMC1 ---> AGRN**	Regulation	info:pmid/10581249#body:109
**PHB --+> GSTP1**	Expression	info:pmid/17135366#abs:5
**PHB --+> GSTP1**	Expression	info:pmid/17324931#body:69
**FN1 ---> GNB2L1**	Regulation	info:pmid/20352103#body:254
**AGT --+> CYR61**	Expression	info:pmid/17234971#abs:7
**AGT --+> CYR61**	Expression	info:pmid/12105167#body:69
**AGT --+> CYR61**	Expression	info:pmid/17307969#body:63
**TXN ---> HSPE1**	Regulation	info:pmid/7876079#body:50
**GPI --+> MMP2**	MolTransport	info:pmid/14968121#body:200
**AGT --+> LGALS3**	Expression	info:pmid/22844495#abs:10
**AGT --+> LGALS3**	Expression	info:pmid/23117656#cont:46
**AGT ---> AGRN**	Expression	info:pmid/14694155#body:243
**GSN ---| TF**	MolTransport	info:pmid/16511569#body:200
**GALNS ---| AGRN**	Expression	info:pmid/12773545#body:249
**PSAP --+> ALB**	MolTransport	info:pmid/10818106#body:286
**AGT ---> PDE4D**	Expression	info:pmid/21151982#body:244
**AGT ---> PDE4D**	Expression	info:pmid/10851231#body:315
**ALB --+> TIMP1**	Expression	info:pmid/22507553#abs:15
**ALB --+> TIMP1**	Expression	info:pmid/14654729#abs:4
**ALB --+> TIMP1**	Expression	info:pmid/16807361#body:201
**ALB --+> TIMP1**	Expression	info:pmid/17369283#body:199
**CTSB ---> ANGPT2**	Expression	info:pmid/21347260#body:133
**TF ---> SOD3**	Regulation	info:pmid/22548386#cont:115
**TF ---> SOD2**	Regulation	info:pmid/22548386#cont:115
**LCN2 --+> SOD2**	Regulation	info:pmid/19904630#abs:10
**QSOX1 ---> MMP2**	Regulation	info:pmid/21989104#abs:8
**QSOX1 ---> MMP2**	Regulation	info:pmid/23098186#cont:445
**QSOX1 ---> MMP2**	Regulation	info:pmid/23680167#cont:17
**PSAP --+> MMP2**	Regulation	info:pmid/21943334#cont:196
**AIMP1 ---> CFL1**	Regulation	info:pmid/22531886#abs:5
**AIMP1 ---> CFL1**	Regulation	info:pmid/21647708#cont:182
**NNMT --+> MMP2**	Expression	info:pmid/21045016#abs:6
**NNMT --+> MMP2**	Expression	info:pmid/23321672#cont:469
**NNMT --+> MMP2**	Expression	info:pmid/23764850#cont:25
**LCN2 ---| TF**	MolTransport	info:pmid/22084236#abs:9
**TXN ---| C3**	Regulation	info:doi/10.1007/82-2012-277#cont:246
**SPP1 ---> LGALS3**	MolTransport	info:pmid/22547442#cont:620
**PHB --+> MMP2**	Regulation	info:pmid/22410782#cont:570
**TCF3 --+> ANGPT2**	Regulation	info:pmid/22792348#cont:323
**SOD3 ---| SPP1**	Regulation	info:pmid/23241403#cont:222
**GNB2L1 ---> MMP2**	Expression	info:pmid/22207523#cont:209
**SPARC --+> CTSB**	Expression	info:pmid/20339379#cont:226
**SPARC --+> CTSB**	Expression	info:pmid/21850018#cont:50
**PRDX4 --+> ALB**	MolTransport	info:pmid/22496424#cont:130
**TXN ---| SOD3**	Expression	info:pmid/20393169#cont:187
**IGFBP7 ---| CYR61**	Regulation	info:pmid/21338521#cont:1028
**SPP1 --+> CYR61**	Expression	info:pmid/22481923#cont:345
**SPARC ---| LGALS3**	Regulation	info:pmid/23467362#cont:286
**LDHA --+> MMP2**	Expression	info:pmid/24131935#cont:125

<table>
<tr><td>SPARC ---| TIMP1</td><td>Expression</td><td>info:pmid/23516489#cont:625</td></tr>
<tr><td>SPARC ---| SOD2</td><td>Expression</td><td>info:pmid/21685937#cont:189</td></tr>
<tr><td>C3 --+> AGT</td><td>Expression</td><td>info:pmid/22089112#abs:10</td></tr>
<tr><td>MMP2 ---| SERPINC1</td><td>Regulation</td><td>info:pmid/1651920#title:1</td></tr>
<tr><td>AGT ---| HP</td><td>Regulation</td><td>info:pmid/20643827#abs:5</td></tr>
<tr><td>AGT ---| HP</td><td>Regulation</td><td>info:pmid/20974581#abs:3</td></tr>
<tr><td>IGFBP7 --+> FN1</td><td>Expression</td><td>info:pmid/22321149#abs:6</td></tr>
<tr><td>IGFBP7 --+> FN1</td><td>Expression</td><td>info:pmid/24373620#abs:7</td></tr>
<tr><td>SOD2 --+> GSTP1</td><td>Expression</td><td>info:pmid/24068538#abs:5</td></tr>
<tr><td>SOD2 ---| COL1A2</td><td>Expression</td><td>info:pmid/10498651#abs:6</td></tr>
<tr><td>SPP1 ---> COL1A2</td><td>Expression</td><td>info:pmid/21514415#abs:5</td></tr>
<tr><td>AGT ---> SERPINC1</td><td>Expression</td><td>info:pmid/10691100#abs:5</td></tr>
<tr><td>AGT ---> SERPINC1</td><td>Expression</td><td>info:pmid/7752579#abs:6</td></tr>
<tr><td>SPARC ---> CFL1</td><td>Regulation</td><td>info:pmid/21798346#abs:10</td></tr>
<tr><td>SERPINC1 ---| C3</td><td>Regulation</td><td>info:pmid/23089609#abs:7</td></tr>
<tr><td>FN1 --+> QSOX1</td><td>MolTransport</td><td>info:pmid/19093006#abs:9</td></tr>
<tr><td>PEBP1 ---| CTSB</td><td>Regulation</td><td>info:pmid/22295570#abs:4</td></tr>
<tr><td>HSPE1 ---> CFL1</td><td>Regulation</td><td>info:pmid/19142874#abs:8</td></tr>
<tr><td>TXN --+> CTSB</td><td>Regulation</td><td>info:pmid/10447715#abs:6</td></tr>
<tr><td>XDH ---> CP</td><td>Regulation</td><td>info:pmid/3755593#abs:1</td></tr>
<tr><td>FN1 ---| SOD3</td><td>Expression</td><td>info:pmid/15754761#abs:8</td></tr>
<tr><td>AGT ---> PXDN</td><td>Regulation</td><td>info:pmid/21292788#abs:6</td></tr>
</table>

Relations include Expression (--->), Inhibition (---|), and Upregulation (--+>).
